# Supplementary material for: Artificial intelligence-enabled electrocardiography from scientific research to clinical application
Source: EMBO Mol Med. 2025 Dec 1;18(1):22–40. doi: 10.1038/s44321-025-00351-y (PMC12808761; doi:10.1038/s44321-025-00351-y)
Supplement: Supplementary file 2 — Appendix [file 44321_2025_351_MOESM2_ESM.pdf]

Table of content

Appendix – AI-ECG clinical trial literature search and study selection 2
References 3

Appendix – AI-ECG clinical trial literature search and study selection

We performed a systematic literature search at 31 Aug 2025 using the keywords including "Randomized controlled trial", "RCT", "Prospective study", "Artificial intelligence", "AI", "Deep learning", "Machine learning", "ECG", "Electrocardiography", and "Electrocardiogram". The primary objective was to identify clinical trials of AI-ECG, with preference for randomized controlled trials (RCTs) but also including studies with prospective, multi-arm designs.

The inclusion and exclusion criteria were defined a priori. Studies were excluded if they: (1) involved non-human samples, reviews, or drug trials; (2) focused only on a small number of ECG features associated with disease outcomes; (3) used models based solely on categorical ECG interpretation results (e.g., “normal/abnormal” classification) without feature-level extraction. Eligible studies included those applying deep learning directly to raw ECG signals, or those using extracted ECG features (e.g., heart rate, PR interval, HRV) for subsequent modeling. We also accepted multimodal models that incorporated ECG data along with additional clinical features.

As shown in Appendix Figure 1, after screening titles and abstracts, 350 of 482 records were excluded.[1-352] Full-text review was performed on the remaining 130 articles. Of these, 64 were not prospective in design,[353-416] 45 were prospective development or accuracy evaluation studies,[417-461] 4 lacked an appropriate control group,[462-465] 5 were published protocols without results,[466-470] and 1 lacked clinical-related endpoints.[471] Ultimately, 11 studies fulfilled all eligibility criteria and were included in the final systematic review.[471-482] Among these, 6 were RCTs,[472-477] 3 were prospective non-RCT studies,[478-480] and 2 were post-hoc analyses of previous RCTs.[481,482]

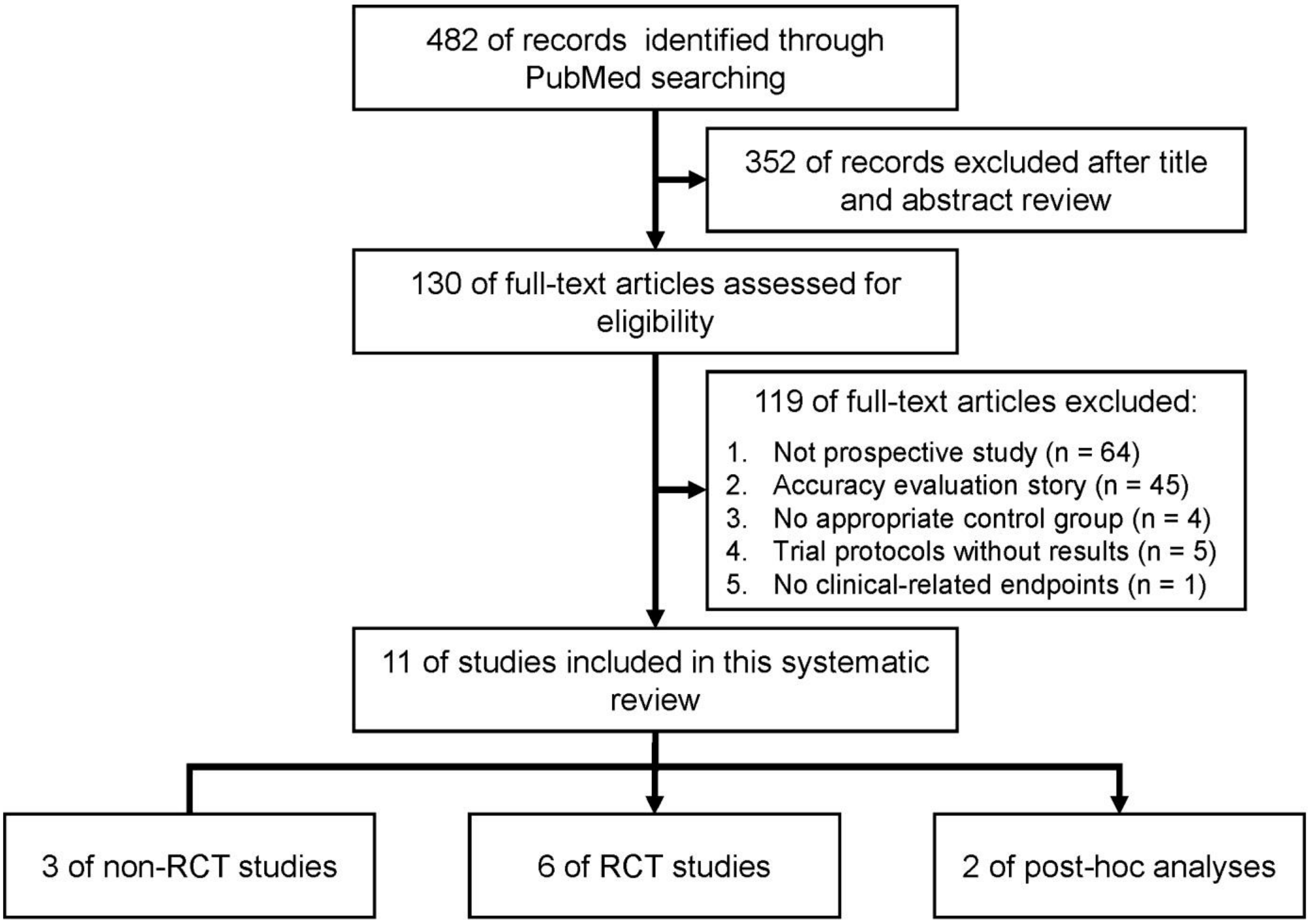

Appendix Figure 1 | PRISMA flow diagram of study selection for AI-ECG clinical trials.

References

1. Kingsley, B.P., Vaughan, M.S. & Vaughan, R.W. Cardiovascular effects of nondepolarizing relaxants employed for pretreatment prior to succinylcholine. *Can Anaesth Soc J* **31**, 13-19 (1984).
2. Maguire, J.H., *et al.* Cardiac morbidity and mortality due to Chagas' disease: prospective electrocardiographic study of a Brazilian community. *Circulation* **75**, 1140-1145 (1987).
3. Välimäki, I.A., Nieminen, T., Antila, K.J. & Southall, D.P. Heart-rate variability and SIDS. Examination of heart-rate patterns using an expert system generator. *Annals of the New York Academy of Sciences* **533**, 228-237 (1988).
4. Nakashima, M., Uematsu, T., Takiguchi, Y., Mizuno, A. & Kanamaru, M. Phase I study of Y-20811, a new long-acting thromboxane synthetase inhibitor by oral administration. *J Clin Pharmacol* **29**, 568-576 (1989).
5. Dukuly, Z.D., *et al.* A prospective study in high risk subjects of electrocardiographic changes with ivermectin. *Trop Med Parasitol* **41**, 73-74 (1990).
6. Miall, W.E. Beta-blockers vs. thiazides in the treatment of hypertension: a review of the experience of the large national trials. *J Cardiovasc Pharmacol* **16 Suppl 5**, S58-63 (1990).
7. Mota, E.A., *et al.* A nine year prospective study of Chagas' disease in a defined rural population in northeast Brazil. *Am J Trop Med Hyg* **42**, 429-440 (1990).
8. Berman, E.J., *et al.* Right ventricular hypertrophy detected by echocardiography in patients with newly diagnosed obstructive sleep apnea. *Chest* **100**, 347-350 (1991).
9. Dash, H.H., Bithal, P.K., Joshi, S. & Saini, S.S. Airway pressure monitoring as an aid in the diagnosis of air embolism. *J Neurosurg Anesthesiol* **5**, 159-163 (1993).
10. Honig, P.K., *et al.* Effect of concomitant administration of cimetidine and ranitidine on the pharmacokinetics and electrocardiographic effects of terfenadine. *Eur J Clin Pharmacol* **45**, 41-46 (1993).
11. Otsuji, Y., *et al.* Influence of left ventricular filling profile during preceding control beats on the occurrence of pulse deficit caused by ventricular premature contractions. *European heart journal* **14**, 1044-1049 (1993).
12. Colì, A., *et al.* [Hypotension controlled with ATP in orthopedic surgery: incidence of atrio-ventricular conduction disorders]. *Minerva Anestesiol* **60**, 21-27 (1994).
13. Fioretti, P.M., *et al.* Atropine increases the accuracy of dobutamine stress echocardiography in patients taking beta-blockers. *European heart journal* **15**, 355-360 (1994).
14. Otsuji, Y., *et al.* Influence of left ventricular filling profile during preceding control beats on pulse pressure during ventricular premature contractions. *European heart journal* **15**, 462-467 (1994).
15. Boden, W.E., *et al.* Design of a placebo-controlled clinical trial of long-acting diltiazem and aspirin versus aspirin alone in patients receiving thrombolysis with a first acute myocardial infarction. Incomplete Infarction Trial of European Research Collaborators Evaluating Prognosis Post-Thrombolysis (diltiazem) (INTERCEPT) Research Group. *The American journal of cardiology* **75**, 1120-1123 (1995).
16. Brannan, M.D., *et al.* Loratadine administered concomitantly with erythromycin: pharmacokinetic and electrocardiographic evaluations. *Clin Pharmacol Ther* **58**, 269-278 (1995).
17. Franco, A.S., *et al.* [Acute cerebrovascular accident and heart disease. Prospective study of 248 patients]. *Rev Port Cardiol* **14**, 291-300, 283 (1995).
18. Regitz-Zagrosek, V., Leuchs, B., Krülls-Münch, J. & Fleck, E. Angiotensin-converting enzyme inhibitors and beta-blockers in long-term treatment of dilated cardiomyopathy. *American heart journal* **129**, 754-761 (1995).
19. Seidl, K., *et al.* [Ventricular arrhythmias after myocardial infarction: risk classification and treatment concepts].

Dtsch Med Wochenschr **120**, 656-660 (1995).
20. Selker, H.P., Griffith, J.L., Patil, S., Long, W.J. & D'Agostino, R.B. A comparison of performance of mathematical predictive methods for medical diagnosis: identifying acute cardiac ischemia among emergency department patients. *J Investig Med* **43**, 468-476 (1995).
21. Stremski, E.S., Brady, W.B., Prasad, K. & Hennes, H.A. Pediatric carbamazepine intoxication. *Annals of emergency medicine* **25**, 624-630 (1995).
22. Yeager, R.A.*, et al.* Reducing perioperative myocardial infarction following vascular surgery. The potential role of beta-blockade. *Arch Surg* **130**, 869-872; discussion 872-863 (1995).
23. Mendall, M.A., Patel, P., Ballam, L., Strachan, D. & Northfield, T.C. C reactive protein and its relation to cardiovascular risk factors: a population based cross sectional study. *BMJ (Clinical research ed.)* **312**, 1061-1065 (1996).
24. Schulman, S.P.*, et al.* Effects of integrelin, a platelet glycoprotein IIb/IIIa receptor antagonist, in unstable angina. A randomized multicenter trial. *Circulation* **94**, 2083-2089 (1996).
25. Andersen, K. & Dellborg, M. Heparin is more effective than inogatran, a low-molecular weight thrombin inhibitor in suppressing ischemia and recurrent angina in unstable coronary disease. Thrombin Inhibition in Myocardial Ischemia (TRIM) Study Group. *The American journal of cardiology* **81**, 939-944 (1998).
26. Braunwald, E.*, et al.* Rationale and clinical evidence for the use of GP IIb/IIIa inhibitors in acute coronary syndromes. *American heart journal* **135**, S56-66 (1998).
27. Degre, S.*, et al.* Effects of spironolactone-altizide on left ventricular hypertrophy. *Acta Cardiol* **53**, 261-267 (1998).
28. Mann, J. & Julius, S. The Valsartan Antihypertensive Long-term Use Evaluation (VALUE) trial of cardiovascular events in hypertension. Rationale and design. *Blood pressure* **7**, 176-183 (1998).
29. Marzaloni, M.*, et al.* [AI-CARE: a multicentric study on unstable angina. Methodology and preliminary data of a project on the improvement of health care quality in Emilia-Romagna]. *G Ital Cardiol* **28**, 1072-1082 (1998).
30. Blatt, S.D.*, et al.* Sudden infant death syndrome, child sexual abuse, and child development. *Curr Opin Pediatr* **11**, 175-186 (1999).
31. Brabant, S.M., Bertrand, M., Eyraud, D., Darmon, P.L. & Coriat, P. The hemodynamic effects of anesthetic induction in vascular surgical patients chronically treated with angiotensin II receptor antagonists. *Anesth Analg* **89**, 1388-1392 (1999).
32. Charoenpan, P.*, et al.* Sleep apnoea syndrome in Ramathibodi Hospital: clinical and polysomnographic baseline data. *Respirology* **4**, 371-374 (1999).
33. Goldstein, B.*, et al.* Effect of N(G)-nitro-L-arginine methyl ester on autonomic modulation of heart rate variability during hypovolemic shock. *Critical care medicine* **27**, 2239-2245 (1999).
34. Hyde, T.A.*, et al.* Four-year survival of patients with acute coronary syndromes without ST-segment elevation and prognostic significance of 0.5-mm ST-segment depression. *The American journal of cardiology* **84**, 379-385 (1999).
35. Jordaens, L. & Tavernier, R. [Survival after myocardial infarction in the nineties. Results of a prospective registry and their implications as to identification of an increased risk of death and especially sudden death]. *J Med Liban* **47**, 181-189 (1999).
36. Mahaffey, K.W.*, et al.* Stroke in patients with acute coronary syndromes: incidence and outcomes in the platelet glycoprotein IIb/IIIa in unstable angina. Receptor suppression using integrilin therapy (PURSUIT) trial. The PURSUIT Investigators. *Circulation* **99**, 2371-2377 (1999).

37. Querfeld, U., *et al.* Probucol for treatment of hyperlipidemia in persistent childhood nephrotic syndrome. Report of a prospective uncontrolled multicenter study. *Pediatric nephrology (Berlin, Germany)* **13**, 7-12 (1999).
38. Robbins, M.A., *et al.* Chest pain--a strong predictor of adverse cardiac events following precutaneous intervention (from the Evaluation of Platelet IIb/IIIa Inhibitor for Stenting Trial [EPISENT])]. *The American journal of cardiology* **84**, 1350-1353, a1358 (1999).
39. Wagner, S., *et al.* [Acute myocardial infarction in Germany between 1996 and 1998: therapy and intrahospital course. Results of the Myocardial Infarction Registry (MIR) in Germany]. *Z Kardiol* **88**, 857-867 (1999).
40. Wung, S.F. & Drew, B. Comparison of 18-lead ECG and selected body surface potential mapping leads in determining maximally deviated ST lead and efficacy in detecting acute myocardial ischemia during coronary occlusion. *Journal of electrocardiology* **32 Suppl**, 30-37 (1999).
41. Hasdai, D., *et al.* Age and outcome after acute coronary syndromes without persistent ST-segment elevation. *American heart journal* **139**, 858-866 (2000).
42. Stöllberger, C., *et al.* Multivariate analysis-based prediction rule for pulmonary embolism. *Thromb Res* **97**, 267-273 (2000).
43. Wasek, W., *et al.* Susceptibility to neuromediated syncope after acute myocardial infarction. *European journal of clinical investigation* **30**, 383-388 (2000).
44. Bellotti, P., *et al.* Specialty-related differences in the epidemiology, clinical profile, management and outcome of patients hospitalized for heart failure; the OSCUR study. Oucome dello Scompenso Cardiaco in relazione all'Utilizzo delle Risore. *European heart journal* **22**, 596-604 (2001).
45. Iwase, M., *et al.* Cardiac functional and structural alterations induced by endotoxin in rats: importance of platelet-activating factor. *Critical care medicine* **29**, 609-617 (2001).
46. Losay, J., *et al.* Late outcome after arterial switch operation for transposition of the great arteries. *Circulation* **104**, I121-126 (2001).
47. Newby, L.K., *et al.* Benefit of glycoprotein IIb/IIIa inhibition in patients with acute coronary syndromes and troponin t-positive status: the paragon-B troponin T substudy. *Circulation* **103**, 2891-2896 (2001).
48. Tapanainen, J.M., Still, A.M., Airaksinen, K.E. & Huikuri, H.V. Prognostic significance of risk stratifiers of mortality, including T wave alternans, after acute myocardial infarction: results of a prospective follow-up study. *Journal of cardiovascular electrophysiology* **12**, 645-652 (2001).
49. Zeymer, U., *et al.* The Na(+)/H(+) exchange inhibitor eniporide as an adjunct to early reperfusion therapy for acute myocardial infarction. Results of the evaluation of the safety and cardioprotective effects of eniporide in acute myocardial infarction (ESCAMI) trial. *Journal of the American College of Cardiology* **38**, 1644-1650 (2001).
50. Altman, R., *et al.* Efficacy assessment of meloxicam, a preferential cyclooxygenase-2 inhibitor, in acute coronary syndromes without ST-segment elevation: the Nonsteroidal Anti-Inflammatory Drugs in Unstable Angina Treatment-2 (NUT-2) pilot study. *Circulation* **106**, 191-195 (2002).
51. Ambrosioni, E. [Management of hypertensive patients with left ventricular hypertrophy]. *Presse Med* **31 Spec No 2**, S13-16 (2002).
52. Hintze, U., Vach, W., Burchardt, H., Videbaek, J. & Møller, M. QT interval dynamics predict mortality in high-risk patients after myocardial infarction. *Scand Cardiovasc J* **36**, 276-281 (2002).
53. Kovar, D., Canto, J.G. & Rogers, W.J. Safety and effectiveness of combined low molecular weight heparin and glycoprotein IIb/IIIa inhibitors. *The American journal of cardiology* **90**, 911-915 (2002).
54. Rashed, W., *et al.* Patient characteristics and practice patterns in the treatment of acute myocardial infarction in

Kuwait: a pilot study. *Medical principles and practice : international journal of the Kuwait University, Health Science Centre* **11**, 196-201 (2002).
55. Thomas, J.T.*, et al.* Utility of history, physical examination, electrocardiogram, and chest radiograph for differentiating normal from decreased systolic function in patients with heart failure. *The American journal of medicine* **112**, 437-445 (2002).
56. Treatment modalities of non-ST-elevation acute coronary syndromes in the real world. Results of the prospective R.OS.A.I.-2 registry. *Ital Heart J* **4**, 782-790 (2003).
57. Bakhai, A.*, et al.* National economic impact of tirofiban for unstable angina and myocardial infarction without ST elevation; example from the United Kingdom. *International journal of cardiology* **91**, 163-172 (2003).
58. Boccara, G.*, et al.* Terlipressin versus norepinephrine to correct refractory arterial hypotension after general anesthesia in patients chronically treated with renin-angiotensin system inhibitors. *Anesthesiology* **98**, 1338-1344 (2003).
59. Bosch, X.*, et al.* [Clinical characteristics, prognosis, and variability in the management of non-ST-segment elevation acute coronary syndromes. Data from the PEPA registry]. *Rev Esp Cardiol* **56**, 346-353 (2003).
60. Ciçek, D.*, et al.* Predictive value of P-wave signal-averaged electrocardiogram for atrial fibrillation in acute myocardial infarction. *Annals of noninvasive electrocardiology : the official journal of the International Society for Holter and Noninvasive Electrocardiology, Inc* **8**, 233-237 (2003).
61. Drögemüller, A.*, et al.* Prognostic value of non-sustained ventricular tachycardias after acute myocardial infarction in the thrombolytic era: importance of combination with frequent ventricular premature beats. *Z Kardiol* **92**, 164-172 (2003).
62. Fesmire, F.M., Peterson, E.D., Roe, M.T. & Wojcik, J.F. Early use of glycoprotein IIb/IIIa inhibitors in the ED treatment of non-ST-segment elevation acute coronary syndromes: a local quality improvement initiative. *The American journal of emergency medicine* **21**, 302-308 (2003).
63. Freestone, B., Rajaratnam, R., Hussain, N. & Lip, G.Y. Admissions with atrial fibrillation in a multiracial population in Kuala Lumpur, Malaysia. *International journal of cardiology* **91**, 233-238 (2003).
64. Heusser, K., Vitkovsky, J., Schmieder, R.E. & Schobel, H.P. AT1 antagonism by eprosartan lowers heart rate variability and baroreflex gain. *Auton Neurosci* **107**, 45-51 (2003).
65. Jansens, J.L., Jottrand, M., Preumont, N., Stoupel, E. & de Cannière, D. Robotic-enhanced biventricular resynchronization: an alternative to endovenous cardiac resynchronization therapy in chronic heart failure. *Ann Thorac Surg* **76**, 413-417; discussion 417 (2003).
66. Okmen, E., Gurol, T., Erdinler, I., Sanli, A. & Cam, N. New-onset conduction defects and their relationship with in-hospital major cardiac events in unstable angina pectoris. *Coron Artery Dis* **14**, 521-525 (2003).
67. Pavesi, P.C.*, et al.* [Epidemiology of acute coronary syndromes in cardiology departments of the Emilia Romagna region: the AI-CARE2 study]. *Ital Heart J Suppl* **4**, 733-744 (2003).
68. Vikman, S.*, et al.* Gap between guidelines and management of patients with acute coronary syndrome without persistent ST elevation. Finnish prospective follow-up survey. *Scand Cardiovasc J* **37**, 187-192 (2003).
69. Wilson, L.D. & Shelat, C. Electrophysiologic and hemodynamic effects of sodium bicarbonate in a canine model of severe cocaine intoxication. *J Toxicol Clin Toxicol* **41**, 777-788 (2003).
70. Zahger, D.*, et al.* Characteristics, management and outcome of patients with prior coronary bypass surgery presenting with acute myocardial infarction. *Cardiology* **99**, 105-110 (2003).
71. Corbalán, R.*, et al.* [Results of the first Chilean registry of unstable angina. Clinical features, risk profile and

treatment]. *Rev Med Chil* **132**, 135-143 (2004).
72. Glass, T.F.*, et al.* Use of artificial intelligence to identify cardiovascular compromise in a model of hemorrhagic shock. *Critical care medicine* **32**, 450-456 (2004).
73. Hiestand, B.C.*, et al.* Insurance status and the treatment of myocardial infarction at academic centers. *Academic emergency medicine : official journal of the Society for Academic Emergency Medicine* **11**, 343-348 (2004).
74. Kanopskiĭ, S.G., Staritskiĭ, A.G. & Bozhko, A.A. [Correction of baroreflex sensitivity impairment and efficacy of prevention of sudden arrhythmic death in patients with postinfarction left ventricular dysfunction]. *Kardiologiia* **44**, 13-18 (2004).
75. Martínez-Ríos, M.A.*, et al.* Comparison of reperfusion regimens with or without tirofiban in ST-elevation acute myocardial infarction. *The American journal of cardiology* **93**, 280-287 (2004).
76. Nieuwlaat, R.*, et al.* [Treatment of patients with acute coronary syndromes in the Netherlands in 2000-2001; a comparison with other European countries and with the guidelines]. *Ned Tijdschr Geneeskd* **148**, 1878-1882 (2004).
77. Ruzyllo, W., Ponikowski, P. & Wilkins, A. Clinical characteristics and methods of treatment of patients with stable coronary heart disease in the primary care settings--the results of the Polish, Multicentre Angina Treatment Pattern (ATP) study. *Int J Clin Pract* **58**, 1127-1133 (2004).
78. Yavuz, T.*, et al.* Role of endogenous adenosine in atrial fibrillation after coronary artery bypass graft. *Clin Cardiol* **27**, 343-346 (2004).
79. Alexander, K.P.*, et al.* Excess dosing of antiplatelet and antithrombin agents in the treatment of non-ST-segment elevation acute coronary syndromes. *Jama* **294**, 3108-3116 (2005).
80. Arslanagic, A., Zulic, I. & Bajraktarevic, A. Clinical study on safety and efficacy of the administration of amlodipine in a combination with lisinopril in hypertensive patients. *Med Arh* **59**, 346-348 (2005).
81. Fuenmayor, A.J., Moreno, G., Landaeta, A. & Fuenmayor, A.M. Inter-atrial conduction time shortens after blood pressure control in hypertensive patients with left ventricular hypertrophy. *International journal of cardiology* **102**, 443-446 (2005).
82. Giugliano, R.P.*, et al.* The early glycoprotein IIb/IIIa inhibition in non-ST-segment elevation acute coronary syndrome (EARLY ACS) trial: a randomized placebo-controlled trial evaluating the clinical benefits of early front-loaded eptifibatide in the treatment of patients with non-ST-segment elevation acute coronary syndrome--study design and rationale. *American heart journal* **149**, 994-1002 (2005).
83. Lefèvre, T.*, et al.* X-sizer for thrombectomy in acute myocardial infarction improves ST-segment resolution: results of the X-sizer in AMI for negligible embolization and optimal ST resolution (X AMINE ST) trial. *Journal of the American College of Cardiology* **46**, 246-252 (2005).
84. Morelli, A.*, et al.* Terlipressin versus norepinephrine to counteract anesthesia-induced hypotension in patients treated with renin-angiotensin system inhibitors: effects on systemic and regional hemodynamics. *Anesthesiology* **102**, 12-19 (2005).
85. Oltrona, L.*, et al.* [The early management of ST-elevation acute myocardial infarction in the Lombardy Region (GestIMA)]. *Ital Heart J Suppl* **6**, 489-497 (2005).
86. Owada, S.*, et al.* "V-H-A Pattern" as a criterion for the differential diagnosis of atypical AV nodal reentrant tachycardia from AV reciprocating tachycardia. *Pacing Clin Electrophysiol* **28**, 667-674 (2005).
87. Pinto, D.S.*, et al.* Administration of intracoronary eptifibatide during ST-elevation myocardial infarction. *The American journal of cardiology* **96**, 1494-1497 (2005).

88. Schachner, T., *et al.* Does preoperative multislice computed tomography predict operative times in total endoscopic coronary artery bypass grafting? *Heart Surg Forum* **8**, E314-318 (2005).
89. Tan, H.Q., Zhu, J., Liang, Y., Zhang, Y. & Liu, L.S. [Two year follow-up of acute ischemic syndrome without ST elevation]. *Zhonghua Yi Xue Za Zhi* **85**, 184-188 (2005).
90. Williams, S.G., *et al.* Does treatment with ACE inhibitors or angiotensin II receptor antagonists prevent atrial fibrillation after dual chamber pacemaker implantation? *Europace : European pacing, arrhythmias, and cardiac electrophysiology : journal of the working groups on cardiac pacing, arrhythmias, and cardiac cellular electrophysiology of the European Society of Cardiology* **7**, 554-559 (2005).
91. Adams, G.L., *et al.* Effectiveness of prehospital wireless transmission of electrocardiograms to a cardiologist via hand-held device for patients with acute myocardial infarction (from the Timely Intervention in Myocardial Emergency, NorthEast Experience [TIME-NE]). *The American journal of cardiology* **98**, 1160-1164 (2006).
92. Arhan, A., *et al.* Assessment of early administration of abciximab in acute ST-segment elevation myocardial infarction in the emergency room. *Presse Med* **35**, 45-50 (2006).
93. Busti, A.J., *et al.* A prospective evaluation of the effect of atazanavir on the QTc interval and QTc dispersion in HIV-positive patients. *HIV Med* **7**, 317-322 (2006).
94. Danzi, G.B., *et al.* Downstream administration of a high-dose tirofiban bolus in high-risk patients with unstable angina undergoing early percutaneous coronary intervention. *International journal of cardiology* **107**, 241-246 (2006).
95. Fernandes, J.L., *et al.* Regression of coronary artery outward remodeling in patients with non-ST-segment acute coronary syndromes: a longitudinal study using noninvasive magnetic resonance imaging. *American heart journal* **152**, 1123-1132 (2006).
96. Hirakawa, Y., *et al.* Age differences in the delivery of cardiac management to women versus men with acute myocardial infarction: an evaluation of the TAMIS-II data. *Int Heart J* **47**, 209-217 (2006).
97. Pokrovskiĭ, A.V., *et al.* [Estimation of the protective effect of Nebilet for perioperative cardiac complications in patients underwent vascular surgery: results of prospective study]. *Angiol Sosud Khir* **12**, 35-41 (2006).
98. Schimrigk, S., *et al.* Oral fumaric acid esters for the treatment of active multiple sclerosis: an open-label, baseline-controlled pilot study. *European journal of neurology* **13**, 604-610 (2006).
99. Schuler, J., Maier, B., Behrens, S. & Thimme, W. Present treatment of acute myocardial infarction in patients over 75 years--data from the Berlin Myocardial Infarction Registry (BHIR). *Clin Res Cardiol* **95**, 360-367 (2006).
100. Svilaas, T., van der Horst, I.C. & Zijlstra, F. Thrombus Aspiration during Percutaneous coronary intervention in Acute myocardial infarction Study (TAPAS)--study design. *American heart journal* **151**, 597.e591-597.e597 (2006).
101. Thiele, H., *et al.* ST-segment recovery and prognosis in patients with ST-elevation myocardial infarction reperfused by prehospital combination fibrinolysis, prehospital initiated facilitated percutaneous coronary intervention, or primary percutaneous coronary intervention. *The American journal of cardiology* **98**, 1132-1139 (2006).
102. Apte, R.S., *et al.* Pegaptanib 1-year systemic safety results from a safety-pharmacokinetic trial in patients with neovascular age-related macular degeneration. *Ophthalmology* **114**, 1702-1712 (2007).
103. Cambou, J.P., Simon, T., Mulak, G., Bataille, V. & Danchin, N. The French registry of Acute ST elevation or non-ST-elevation Myocardial Infarction (FAST-MI): study design and baseline characteristics. *Arch Mal Coeur Vaiss* **100**, 524-534 (2007).
104. Christoforidis, A., *et al.* Four-year evaluation of myocardial and liver iron assessed prospectively with serial MRI

scans in young patients with beta-thalassaemia major: comparison between different chelation regimens. *Eur J Haematol* **78**, 52-57 (2007).

105. Eggers, K.M., *et al.* Artificial neural network algorithms for early diagnosis of acute myocardial infarction and prediction of infarct size in chest pain patients. *International journal of cardiology* **114**, 366-374 (2007).

106. Jabara, R., *et al.* Comparison of multidetector 64-slice computed tomographic angiography to coronary angiography to assess the patency of coronary artery bypass grafts. *The American journal of cardiology* **99**, 1529-1534 (2007).

107. Ley, S., *et al.* Evaluation of aortic regurgitation in congenital heart disease: value of MR imaging in comparison to echocardiography. *Pediatr Radiol* **37**, 426-436 (2007).

108. Lin, T.H., *et al.* The C-allele of tissue inhibitor of metalloproteinases 2 is associated with increased magnitude of QT dispersion prolongation in elderly Chinese - 4-year follow-up study. *Clinica chimica acta; international journal of clinical chemistry* **386**, 87-93 (2007).

109. Onat, A., *et al.* [Serum apolipoprotein B is superior to LDL-cholesterol level in predicting incident coronary disease among Turks]. *Anadolu kardiyoloji dergisi : AKD = the Anatolian journal of cardiology* **7**, 128-133 (2007).

110. Rodrigues, A.M., Hueb, M., Nery, A.F. & Fontes, C.J. Possible cardioprotective effect of angiotensin-converting enzyme inhibitors during treatment of American tegumentary leishmaniasis with meglumine antimoniate. *Acta Trop* **102**, 113-118 (2007).

111. Schang, D., Feuilloy, M., Plantier, G., Fortrat, J.O. & Nicolas, P. Early prediction of unexplained syncope by support vector machines. *Physiol Meas* **28**, 185-197 (2007).

112. Aboukhalil, A., Nielsen, L., Saeed, M., Mark, R.G. & Clifford, G.D. Reducing false alarm rates for critical arrhythmias using the arterial blood pressure waveform. *Journal of biomedical informatics* **41**, 442-451 (2008).

113. Chen, J.Y., *et al.* Association of premature ventricular complexes with central aortic pressure indices and pulse wave velocity. *American heart journal* **155**, 500.e501-506 (2008).

114. Clutton, R.E. & Glasby, M.A. Cardiovascular and autonomic nervous effects of edrophonium and atropine combinations during neuromuscular blockade antagonism in sheep. *Vet Anaesth Analg* **35**, 191-200 (2008).

115. Diener, H.C., *et al.* DP-b99, a membrane-activated metal ion chelator, as neuroprotective therapy in ischemic stroke. *Stroke* **39**, 1774-1778 (2008).

116. Dziewierz, A., *et al.* In-hospital management and mortality in elderly patients with non-ST-segment elevation acute coronary syndromes treated in centers without on-site invasive facilities. *Cardiology journal* **15**, 451-457 (2008).

117. Lazzerini, P.E., *et al.* Arrhythmic risk during acute infusion of infliximab: a prospective, single-blind, placebo-controlled, crossover study in patients with chronic arthritis. *J Rheumatol* **35**, 1958-1965 (2008).

118. Middleton, P.M., *et al.* Spectral analysis of finger photoplethysmographic waveform variability in a model of mild to moderate haemorrhage. *J Clin Monit Comput* **22**, 343-353 (2008).

119. Monir, G. & Pollak, S.J. Consistency of the CFAE phenomena using custom software for automated detection of complex fractionated atrial electrograms (CFAEs) in the left atrium during atrial fibrillation. *Journal of cardiovascular electrophysiology* **19**, 915-919 (2008).

120. Prado, T.M., DuBois, W.R., Ko, J.C., Mandsager, R.E. & Morgan, G.L. A comparison of two combinations of xylazine-ketamine administered intramuscularly to alpacas and of reversal with tolazoline. *Vet Anaesth Analg* **35**, 201-207 (2008).

121. Singerman, L.J., *et al.* Pegaptanib sodium for neovascular age-related macular degeneration: third-year safety results of the VEGF Inhibition Study in Ocular Neovascularisation (VISION) trial. *Br J Ophthalmol* **92**, 1606-1611

(2008).
122. Tsou, C.H., *et al.* Clinical assessment of propofol-induced yawning with heart rate variability: a pilot study. *J Clin Anesth* **20**, 25-29 (2008).
123. Tuncer, M., *et al.* [Comparison of effects of nebivolol and atenolol on P-wave dispersion in patients with hypertension]. *Kardiologiia* **48**, 42-45 (2008).
124. Eljamel, M.S. Robotic neurological surgery applications: accuracy and consistency or pure fantasy? *Stereotact Funct Neurosurg* **87**, 88-93 (2009).
125. Koeth, O., *et al.* Clinical benefit of early reperfusion therapy in patients with ST-elevation myocardial infarction usually excluded from randomized clinical trials (results from the Maximal Individual Therapy in Acute Myocardial Infarction Plus [MITRA Plus] registry). *The American journal of cardiology* **104**, 1074-1077 (2009).
126. Nickson, C.P., Waugh, E.B., Jacups, S.P. & Currie, B.J. Irukandji syndrome case series from Australia's Tropical Northern Territory. *Annals of emergency medicine* **54**, 395-403 (2009).
127. Qureshi, G., *et al.* QPV interval as a measure of arterial stiffness in women with systemic lupus erythematosus. *Clin Cardiol* **32**, 154-158 (2009).
128. Rakowski, T., *et al.* Early abciximab administration before transfer for primary percutaneous coronary interventions for ST-elevation myocardial infarction reduces 1-year mortality in patients with high-risk profile. Results from EUROTRANSFER registry. *American heart journal* **158**, 569-575 (2009).
129. Akerblom, A., *et al.* Eptifibatide is noninferior to abciximab in primary percutaneous coronary intervention: results from the SCAAR (Swedish Coronary Angiography and Angioplasty Registry). *Journal of the American College of Cardiology* **56**, 470-475 (2010).
130. Berkowitsch, A., *et al.* Therapy with renin-angiotensin system blockers after pulmonary vein isolation in patients with atrial fibrillation: who is a responder? *Pacing Clin Electrophysiol* **33**, 1101-1111 (2010).
131. Cadena, M., *et al.* Method to observe hemodynamic and metabolic changes during hemodiafiltration therapy with exercise. *Annu Int Conf IEEE Eng Med Biol Soc* **2010**, 1206-1209 (2010).
132. Dabrowski, R., *et al.* Effect of combined spironolactone-β-blocker ± enalapril treatment on occurrence of symptomatic atrial fibrillation episodes in patients with a history of paroxysmal atrial fibrillation (SPIR-AF study). *The American journal of cardiology* **106**, 1609-1614 (2010).
133. Salgado, D.M., *et al.* Heart and skeletal muscle are targets of dengue virus infection. *Pediatr Infect Dis J* **29**, 238-242 (2010).
134. Timmer, J.R., *et al.* Pre-hospital administration of tirofiban in diabetic patients with ST-elevation myocardial infarction undergoing primary angioplasty: a sub-analysis of the On-Time 2 trial. *EuroIntervention : journal of EuroPCR in collaboration with the Working Group on Interventional Cardiology of the European Society of Cardiology* **6**, 336-342 (2010).
135. Zeymer, U., *et al.* Randomized comparison of eptifibatide versus abciximab in primary percutaneous coronary intervention in patients with acute ST-segment elevation myocardial infarction: results of the EVA-AMI Trial. *Journal of the American College of Cardiology* **56**, 463-469 (2010).
136. Zeymer, U., Zahn, R., Senges, J. & Gitt, A. [Registries of myocardial infarction in Germany. Consequences for drug therapy of patients with acute ST elevation myocardial infarction]. *Internist (Berl)* **51**, 1324-1327, 1329 (2010).
137. Briasoulis, A., Agarwal, V. & Pierce, W.J. QT prolongation and torsade de pointes induced by fluoroquinolones: infrequent side effects from commonly used medications. *Cardiology* **120**, 103-110 (2011).
138. Camm, A.J., *et al.* Real-life observations of clinical outcomes with rhythm- and rate-control therapies for atrial

fibrillation RECORDAF (Registry on Cardiac Rhythm Disorders Assessing the Control of Atrial Fibrillation). *Journal of the American College of Cardiology* **58**, 493-501 (2011).
139. Maejima, Y.*, et al.* Synergistic effect of combined HMG-CoA reductase inhibitor and angiotensin-II receptor blocker therapy in patients with chronic heart failure: the HF-COSTAR trial. *Circulation journal : official journal of the Japanese Circulation Society* **75**, 589-595 (2011).
140. Schuller, S., Van Israël, N., Vanbelle, S., Clercx, C. & McEntee, K. Lack of efficacy of low-dose spironolactone as adjunct treatment to conventional congestive heart failure treatment in dogs. *J Vet Pharmacol Ther* **34**, 322-331 (2011).
141. Stone, G.W.*, et al.* Heparin plus a glycoprotein IIb/IIIa inhibitor versus bivalirudin monotherapy and paclitaxel-eluting stents versus bare-metal stents in acute myocardial infarction (HORIZONS-AMI): final 3-year results from a multicentre, randomised controlled trial. *Lancet (London, England)* **377**, 2193-2204 (2011).
142. Bradley, J.*, et al.* Pulse transit time and assessment of childhood sleep disordered breathing. *Arch Otolaryngol Head Neck Surg* **138**, 398-403 (2012).
143. Dedov, D.V., Ivanov, A.P. & Él'gardt, I.A. [Management of arrhythmia in patients with arterial hypertension]. *Klin Med (Mosk)* **90**, 39-42 (2012).
144. Ederhy, S.*, et al.* Frequency and management of troponin I elevation in patients treated with molecular targeted therapies in phase I trials. *Invest New Drugs* **30**, 611-615 (2012).
145. Esquitin, R.*, et al.* Left ventricular hypertrophy by electrocardiography and echocardiography in the African American Study of Kidney Disease Cohort Study. *Journal of the American Society of Hypertension : JASH* **6**, 193-200 (2012).
146. Mentz, R.J.*, et al.* Atrial fibrillation or flutter on initial electrocardiogram is associated with worse outcomes in patients admitted for worsening heart failure with reduced ejection fraction: findings from the EVEREST Trial. *American heart journal* **164**, 884-892.e882 (2012).
147. Nölker, G.*, et al.* Novel robotic catheter manipulation system integrated with remote magnetic navigation for fully remote ablation of atrial tachyarrhythmias: a two-centre evaluation. *Europace : European pacing, arrhythmias, and cardiac electrophysiology : journal of the working groups on cardiac pacing, arrhythmias, and cardiac cellular electrophysiology of the European Society of Cardiology* **14**, 1715-1718 (2012).
148. Ohlmann, P.*, et al.* Prehospital abciximab in ST-segment elevation myocardial infarction: results of the randomized, double-blind MISTRAL study. *Circ Cardiovasc Interv* **5**, 69-76, s61 (2012).
149. Rillig, A.*, et al.* Incidence and long-term follow-up of silent cerebral lesions after pulmonary vein isolation using a remote robotic navigation system as compared with manual ablation. *Circulation. Arrhythmia and electrophysiology* **5**, 15-21 (2012).
150. Shimada, Y.J., Nakra, N.C., Fox, J.T. & Kanei, Y. Meta-analysis of prospective randomized controlled trials comparing intracoronary versus intravenous abciximab in patients with ST-elevation myocardial infarction undergoing primary percutaneous coronary intervention. *The American journal of cardiology* **109**, 624-628 (2012).
151. Singh, H.S.*, et al.* Comparison of abciximab versus eptifibatide during percutaneous coronary intervention in ST-segment elevation myocardial infarction (from the HORIZONS-AMI trial). *The American journal of cardiology* **110**, 940-947 (2012).
152. Steg, P.G.*, et al.* Heart rate and use of beta-blockers in stable outpatients with coronary artery disease. *PloS one* **7**, e36284 (2012).
153. Tsou, C.H.*, et al.* "Altered Short-Term Dynamics of Cardio-Respiratory Interaction during Propofol-Induced

Yawning". *Chin J Physiol* **55**, 169-177 (2012).
154. Varenhorst, C., *et al.* Factors contributing to the lower mortality with ticagrelor compared with clopidogrel in patients undergoing coronary artery bypass surgery. *Journal of the American College of Cardiology* **60**, 1623-1630 (2012).
155. Viollet, L., Thrush, P.T., Flanigan, K.M., Mendell, J.R. & Allen, H.D. Effects of angiotensin-converting enzyme inhibitors and/or beta blockers on the cardiomyopathy in Duchenne muscular dystrophy. *The American journal of cardiology* **110**, 98-102 (2012).
156. Abbate, A., *et al.* Effects of interleukin-1 blockade with anakinra on adverse cardiac remodeling and heart failure after acute myocardial infarction [from the Virginia Commonwealth University-Anakinra Remodeling Trial (2) (VCU-ART2) pilot study]. *The American journal of cardiology* **111**, 1394-1400 (2013).
157. Auriemma, R.S., *et al.* Treatment with GH receptor antagonist in acromegaly: effect on cardiac arrhythmias. *European journal of endocrinology* **168**, 15-22 (2013).
158. Beigel, R., *et al.* Antiplatelet effect of thienopyridine (clopidogrel or prasugrel) pretreatment in patients undergoing primary percutaneous intervention for ST elevation myocardial infarction. *The American journal of cardiology* **112**, 1551-1556 (2013).
159. Caudron, J., *et al.* MR relaxometry and perfusion of the myocardium in spontaneously hypertensive rat: correlation with histopathology and effect of anti-hypertensive therapy. *European radiology* **23**, 1871-1881 (2013).
160. Chen, Y., *et al.* Impact of selective infarct-related artery infusion of tirofiban on myocardial reperfusion and bleeding complications in patients with acute myocardial infarction: the SUIT-AMI trial. *J Invasive Cardiol* **25**, 376-382 (2013).
161. Dawood, F.Z., *et al.* Electrocardiographic spatial QRS-T angle and incident cardiovascular disease in HIV-infected patients (from the Strategies for the Management of Antiretroviral Therapy [SMART] study). *The American journal of cardiology* **111**, 118-124 (2013).
162. Elizaga, M.L., *et al.* Prospective surveillance for cardiac adverse events in healthy adults receiving modified vaccinia Ankara vaccines: a systematic review. *PloS one* **8**, e54407 (2013).
163. Gierlotka, M., *et al.* Outcomes of invasive treatment in very elderly Polish patients with non-ST-segment-elevation myocardial infarction from 2003-2009 (from the PL-ACS registry). *Cardiology journal* **20**, 34-43 (2013).
164. Hacıoğlu, Y., *et al.* Use of cardiac CT angiography imaging in an epidemiology study - the Methodology of the Multicenter AIDS Cohort Study cardiovascular disease substudy. *Anadolu kardiyoloji dergisi : AKD = the Anatolian journal of cardiology* **13**, 207-214 (2013).
165. Janda, M., *et al.* Design and implementation of a control system reflecting the level of analgesia during general anesthesia. *Biomed Tech (Berl)* **58**, 1-11 (2013).
166. Khan, E.M., *et al.* First experience with a novel robotic remote catheter system: Amigo™ mapping trial. *J Interv Card Electrophysiol* **37**, 121-129 (2013).
167. La Fountaine, M.F., Wecht, J.M. & Bauman, W.A. Acute nitric oxide synthase inhibition and cardiac conduction in persons with spinal cord injury: a short report. *Pharmazie* **68**, 245-250 (2013).
168. Saia, F., *et al.* Incidence and outcome of high on-treatment platelet reactivity in patients with non-ST elevation acute coronary syndromes undergoing percutaneous coronary intervention (from the VIP [VerifyNow and Inhibition of Platelet Reactivity] study). *The American journal of cardiology* **112**, 792-798 (2013).
169. Satsumae, T., Yamaguchi, H., Inomata, S. & Tanaka, M. Magnesium sulfate attenuates tourniquet pain in healthy volunteers. *J Anesth* **27**, 231-235 (2013).

170. Soguero-Ruiz, C., *et al.* Ontology for heart rate turbulence domain from the conceptual model of SNOMED-CT. *IEEE transactions on bio-medical engineering* **60**, 1825-1833 (2013).
171. Wimmer, N.J., Scirica, B.M. & Stone, P.H. The clinical significance of continuous ECG (ambulatory ECG or Holter) monitoring of the ST-segment to evaluate ischemia: a review. *Prog Cardiovasc Dis* **56**, 195-202 (2013).
172. Auffret, V., *et al.* Efficacy of pre-hospital use of glycoprotein IIb/IIIa inhibitors in ST-segment elevation myocardial infarction before mechanical reperfusion in a rapid-transfer network (from the Acute Myocardial Infarction Registry of Brittany). *The American journal of cardiology* **114**, 214-223 (2014).
173. Azevedo, L.F., *et al.* Sport modality affects bradycardia level and its mechanisms of control in professional athletes. *Int J Sports Med* **35**, 954-959 (2014).
174. Çetin, S., *et al.* Infliximab, an anti-TNF-alpha agent, improves left atrial abnormalities in patients with rheumatoid arthritis: preliminary results. *Cardiovascular journal of Africa* **25**, 168-175 (2014).
175. Fernández-Rodríguez, D., *et al.* Optimization in stent implantation by manual thrombus aspiration in ST-segment-elevation myocardial infarction: findings from the EXAMINATION trial. *Circ Cardiovasc Interv* **7**, 294-300 (2014).
176. Gloekler, S., *et al.* The effect of heart rate reduction by ivabradine on collateral function in patients with chronic stable coronary artery disease. *Heart (British Cardiac Society)* **100**, 160-166 (2014).
177. Iagaru, A., *et al.* (18)F-FPPRGD2 PET/CT: pilot phase evaluation of breast cancer patients. *Radiology* **273**, 549-559 (2014).
178. Johannesen, L., *et al.* Differentiating drug-induced multichannel block on the electrocardiogram: randomized study of dofetilide, quinidine, ranolazine, and verapamil. *Clin Pharmacol Ther* **96**, 549-558 (2014).
179. Kas'ianenko, V.I., Denisov, N.L. & Vasil'ev Iu, V. [Use of itopride in the symptoms of functional dyspepsia in Russia: results of a phase IV prospective open-label multicenter clinical trial]. *Ter Arkh* **86**, 35-41 (2014).
180. Kauf, T.L., *et al.* Alvimopan, a peripherally acting µ-opioid receptor antagonist, is associated with reduced costs after radical cystectomy: economic analysis of a phase 4 randomized, controlled trial. *J Urol* **191**, 1721-1727 (2014).
181. Statsenko, M.E., Turkina, S.V. & Shilina, N.N. [Role of pFox inhibitors in the treatment of patients with acute myocardial ischemia]. *Ter Arkh* **86**, 54-59 (2014).
182. Stone, S.G., *et al.* Incidence, predictors, and implications of reinfarction after primary percutaneous coronary intervention in ST-segment-elevation myocardial infarction: the Harmonizing Outcomes with Revascularization and Stents in Acute Myocardial Infarction Trial. *Circ Cardiovasc Interv* **7**, 543-551 (2014).
183. Udell, J.A., *et al.* Prasugrel versus clopidogrel in patients with ST-segment elevation myocardial infarction according to timing of percutaneous coronary intervention: a TRITON-TIMI 38 subgroup analysis (Trial to Assess Improvement in Therapeutic Outcomes by Optimizing Platelet Inhibition with Prasugrel-Thrombolysis In Myocardial Infarction 38). *JACC. Cardiovascular interventions* **7**, 604-612 (2014).
184. Valgimigli, M., *et al.* Usefulness and safety of vorapaxar in patients with non-ST-segment elevation acute coronary syndrome undergoing percutaneous coronary intervention (from the TRACER Trial). *The American journal of cardiology* **114**, 665-673 (2014).
185. Whellan, D.J., *et al.* Vorapaxar in acute coronary syndrome patients undergoing coronary artery bypass graft surgery: subgroup analysis from the TRACER trial (Thrombin Receptor Antagonist for Clinical Event Reduction in Acute Coronary Syndrome). *Journal of the American College of Cardiology* **63**, 1048-1057 (2014).
186. de Ville de Goyet, M., *et al.* Prospective cardiac MRI for the analysis of biventricular function in children undergoing cancer treatments. *Pediatr Blood Cancer* **62**, 867-874 (2015).

187. Jobs, A., *et al.* Effect of Pericardial Effusion Complicating ST-Elevation Myocardial Infarction as Predictor of Extensive Myocardial Damage and Prognosis. *The American journal of cardiology* **116**, 1010-1016 (2015).
188. Kovacs, R.J., *et al.* Cardiac Safety of TGF-β Receptor I Kinase Inhibitor LY2157299 Monohydrate in Cancer Patients in a First-in-Human Dose Study. *Cardiovasc Toxicol* **15**, 309-323 (2015).
189. Mathews, R., *et al.* Early Medication Nonadherence After Acute Myocardial Infarction: Insights into Actionable Opportunities From the TReatment with ADP receptor iNhibitorS: Longitudinal Assessment of Treatment Patterns and Events after Acute Coronary Syndrome (TRANSLATE-ACS) Study. *Circulation. Cardiovascular quality and outcomes* **8**, 347-356 (2015).
190. Mejhert, M. & Kahan, T. A management programme for suspected heart failure in primary care in cooperation with specialists in cardiology. *Eur J Gen Pract* **21**, 26-32 (2015).
191. Paisible, A.L., *et al.* HIV infection, cardiovascular disease risk factor profile, and risk for acute myocardial infarction. *J Acquir Immune Defic Syndr* **68**, 209-216 (2015).
192. Pöss, J., *et al.* Left Ventricular Thrombus Formation After ST-Segment-Elevation Myocardial Infarction: Insights From a Cardiac Magnetic Resonance Multicenter Study. *Circ Cardiovasc Imaging* **8**, e003417 (2015).
193. Skyttä, T., Tuohinen, S., Virtanen, V., Raatikainen, P. & Kellokumpu-Lehtinen, P.L. The concurrent use of aromatase inhibitors and radiotherapy induces echocardiographic changes in patients with breast cancer. *Anticancer Res* **35**, 1559-1566 (2015).
194. Song, P.S., *et al.* Comparative Effectiveness of Angiotensin II Receptor Blockers Versus Angiotensin-Converting Enzyme Inhibitors Following Contemporary Treatments in Patients with Acute Myocardial Infarction: Results from the Korean Working Group in Myocardial Infarction (KorMI) Registry. *Am J Cardiovasc Drugs* **15**, 439-449 (2015).
195. Bueno, H., *et al.* Opportunities for improvement in anti-thrombotic therapy and other strategies for the management of acute coronary syndromes: Insights from EPICOR, an international study of current practice patterns. *European heart journal. Acute cardiovascular care* **5**, 3-12 (2016).
196. Cardoso, C.S., *et al.* Longitudinal study of patients with chronic Chagas cardiomyopathy in Brazil (SaMi-Trop project): a cohort profile. *BMJ open* **6**, e011181 (2016).
197. DeBo, R.J., *et al.* Late Effects of Total-Body Gamma Irradiation on Cardiac Structure and Function in Male Rhesus Macaques. *Radiat Res* **186**, 55-64 (2016).
198. Erlinge, D., *et al.* Bivalirudin versus heparin in non-ST and ST-segment elevation myocardial infarction-a registry-based randomized clinical trial in the SWEDEHEART registry (the VALIDATE-SWEDEHEART trial). *American heart journal* **175**, 36-46 (2016).
199. Fakhri, Y., *et al.* Pre-hospital electrocardiographic severity and acuteness scores predict left ventricular function in patients with ST elevation myocardial infarction. *Journal of electrocardiology* **49**, 284-291 (2016).
200. Iyama, Y., *et al.* Low-Contrast and Low-Radiation Dose Protocol in Cardiac Computed Tomography: Usefulness of Low Tube Voltage and Knowledge-Based Iterative Model Reconstruction Algorithm. *J Comput Assist Tomogr* **40**, 941-947 (2016).
201. Johannesen, L., *et al.* Late sodium current block for drug-induced long QT syndrome: Results from a prospective clinical trial. *Clin Pharmacol Ther* **99**, 214-223 (2016).
202. Malebranche, R., Tabou Moyo, C., Morisset, P.H., Raphael, N.A. & Wilentz, J.R. Clinical and echocardiographic characteristics and outcomes in congestive heart failure at the Hospital of The State University of Haiti. *American heart journal* **178**, 151-160 (2016).
203. Turley, S.L., Francis, K.E., Lowe, D.K. & Cahoon, W.D., Jr. Emerging role of ivabradine for rate control in atrial

fibrillation. *Ther Adv Cardiovasc Dis* **10**, 348-352 (2016).

204. Zhang, H.P.*, et al.* Impact of Chronic Total Occlusion in a Noninfarct-related Artery on Clinical Outcomes in Patients With Acute ST-elevation Myocardial Infarction Undergoing Primary Percutaneous Coronary Intervention. *Medicine* **95**, e2441 (2016).

205. Choi, S.Y.*, et al.* Angiotensin-converting enzyme inhibitors versus angiotensin II receptor blockers in acute ST-segment elevation myocardial infarction patients with diabetes mellitus undergoing percutaneous coronary intervention. *International journal of cardiology* **249**, 48-54 (2017).

206. Costantino, G.*, et al.* Neural networks as a tool to predict syncope risk in the Emergency Department. *Europace : European pacing, arrhythmias, and cardiac electrophysiology : journal of the working groups on cardiac pacing, arrhythmias, and cardiac cellular electrophysiology of the European Society of Cardiology* **19**, 1891-1895 (2017).

207. Erne, P.*, et al.* Left bundle-branch block in patients with acute myocardial infarction: Presentation, treatment, and trends in outcome from 1997 to 2016 in routine clinical practice. *American heart journal* **184**, 106-113 (2017).

208. Ferrero-de-Loma-Osorio, Á.*, et al.* Time-to-Effect-Based Dosing Strategy for Cryoballoon Ablation in Patients With Paroxysmal Atrial Fibrillation: Results of the plusONE Multicenter Randomized Controlled Noninferiority Trial. *Circulation. Arrhythmia and electrophysiology* **10**(2017).

209. Kiciński, P.*, et al.* Spatial QRS-T angle in patients with newly diagnosed obstructive sleep apnea syndrome. *Adv Med Sci* **62**, 240-245 (2017).

210. Lee, S.E.*, et al.* Reverse J-Curve Relationship Between On-Treatment Blood Pressure and Mortality in Patients With Heart Failure. *JACC Heart Fail* **5**, 810-819 (2017).

211. Madhavan, M.V.*, et al.* Is routine post-procedural anticoagulation warranted after primary percutaneous coronary intervention in ST-segment elevation myocardial infarction? Insights from the HORIZONS-AMI trial. *European heart journal. Acute cardiovascular care* **6**, 650-658 (2017).

212. Marian, M.J.*, et al.* Ticagrelor and Eptifibatide Bolus Versus Ticagrelor and Eptifibatide Bolus With 2-Hour Infusion in High-Risk Acute Coronary Syndromes Patients Undergoing Early Percutaneous Coronary Intervention. *Journal of the American Heart Association* **6**(2017).

213. Osawa, K.*, et al.* Rationale and design of a randomized trial of apixaban vs warfarin to evaluate atherosclerotic calcification and vulnerable plaque progression. *Clin Cardiol* **40**, 807-813 (2017).

214. Oskouie, S.K., Prenner, S.B., Shah, S.J. & Sauer, A.J. Differences in Repolarization Heterogeneity Among Heart Failure With Preserved Ejection Fraction Phenotypic Subgroups. *The American journal of cardiology* **120**, 601-606 (2017).

215. Cardoso, C.S.*, et al.* Beneficial effects of benznidazole in Chagas disease: NIH SaMi-Trop cohort study. *PLoS Negl Trop Dis* **12**, e0006814 (2018).

216. Chobanyan-Jürgens, K.*, et al.* Cardiac pacemaker channel (HCN4) inhibition and atrial arrhythmogenesis after releasing cardiac sympathetic activation. *Scientific reports* **8**, 7748 (2018).

217. de Diego, C.*, et al.* Effects of angiotensin-neprilysin inhibition compared to angiotensin inhibition on ventricular arrhythmias in reduced ejection fraction patients under continuous remote monitoring of implantable defibrillator devices. *Heart Rhythm* **15**, 395-402 (2018).

218. Hussein, A.*, et al.* Use of Ablation Index-Guided Ablation Results in High Rates of Durable Pulmonary Vein Isolation and Freedom From Arrhythmia in Persistent Atrial Fibrillation Patients: The PRAISE Study Results. *Circulation. Arrhythmia and electrophysiology* **11**, e006576 (2018).

219. Ntusi, N.A.B.*, et al.* Anti-TNF modulation reduces myocardial inflammation and improves cardiovascular function

in systemic rheumatic diseases. *International journal of cardiology* **270**, 253-259 (2018).
220. Sengupta, P.P., Kulkarni, H. & Narula, J. Prediction of Abnormal Myocardial Relaxation From Signal Processed Surface ECG. *Journal of the American College of Cardiology* **71**, 1650-1660 (2018).
221. Shunmugam, S.R.*, et al.* A double-blind, randomised, placebo-controlled, cross-over study assessing the use of XEN-D0103 in patients with paroxysmal atrial fibrillation and implanted pacemakers allowing continuous beat-to-beat monitoring of drug efficacy. *J Interv Card Electrophysiol* **51**, 191-197 (2018).
222. Wallis, R.*, et al.* CiPA challenges and opportunities from a non-clinical, clinical and regulatory perspectives. An overview of the safety pharmacology scientific discussion. *J Pharmacol Toxicol Methods* **93**, 15-25 (2018).
223. Casella, M.*, et al.* Ablation Index as a predictor of long-term efficacy in premature ventricular complex ablation: A regional target value analysis. *Heart Rhythm* **16**, 888-895 (2019).
224. Karaoğuz, M.R.*, et al.* The quality of ECG data acquisition, and diagnostic performance of a novel adhesive patch for ambulatory cardiac rhythm monitoring in arrhythmia detection. *Journal of electrocardiology* **54**, 28-35 (2019).
225. Kwon, S.*, et al.* Deep Learning Approaches to Detect Atrial Fibrillation Using Photoplethysmographic Signals: Algorithms Development Study. *JMIR mHealth and uHealth* **7**, e12770 (2019).
226. Melero-Alegria, J.I.*, et al.* SALMANTICOR study. Rationale and design of a population-based study to identify structural heart disease abnormalities: a spatial and machine learning analysis. *BMJ open* **9**, e024605 (2019).
227. Mulder, M.B.*, et al.* Exercise-Induced Changes in Compensatory Reserve and Heart Rate Complexity. *Aerosp Med Hum Perform* **90**, 1009-1015 (2019).
228. Tamraz, B.*, et al.* Association between Use of Methadone, Other Central Nervous System Depressants, and QTc Interval-Prolonging Medications and Risk of Mortality in a Large Cohort of Women Living with or at Risk for Human Immunodeficiency Virus Infection. *Pharmacotherapy* **39**, 899-911 (2019).
229. Yap, J.*, et al.* Harnessing technology and molecular analysis to understand the development of cardiovascular diseases in Asia: a prospective cohort study (SingHEART). *BMC cardiovascular disorders* **19**, 259 (2019).
230. Diller, G.P.*, et al.* Prediction of prognosis in patients with tetralogy of Fallot based on deep learning imaging analysis. *Heart (British Cardiac Society)* **106**, 1007-1014 (2020).
231. Grün, D.*, et al.* Identifying Heart Failure in ECG Data With Artificial Intelligence-A Meta-Analysis. *Front Digit Health* **2**, 584555 (2020).
232. Guglielmi, V.*, et al.* Mind the Heart: Electrocardiography-gated cardiac computed tomography-angiography in acute ischaemic stroke-rationale and study design. *Eur Stroke J* **5**, 441-448 (2020).
233. Kim, K.H.*, et al.* Emergency department routine data and the diagnosis of acute ischemic heart disease in patients with atypical chest pain. *PloS one* **15**, e0241920 (2020).
234. Kwon, S.*, et al.* Detection of Atrial Fibrillation Using a Ring-Type Wearable Device (CardioTracker) and Deep Learning Analysis of Photoplethysmography Signals: Prospective Observational Proof-of-Concept Study. *Journal of medical Internet research* **22**, e16443 (2020).
235. Li, Z.*, et al.* Risk factors for Keshan disease: a prospective cohort study protocol of gut flora. *BMC cardiovascular disorders* **20**, 481 (2020).
236. Nanasato, M., Matsuo, S., Nakajima, K., Nishimura, S. & Nishimura, T. Predictive value of electrocardiography-gated myocardial perfusion imaging to new-onset heart failure in patients with chronic kidney disease: findings from the J-ACCESS 3 study. *Int J Cardiovasc Imaging* **36**, 749-755 (2020).
237. Orfanoudaki, A.*, et al.* Machine learning provides evidence that stroke risk is not linear: The non-linear Framingham stroke risk score. *PloS one* **15**, e0232414 (2020).

238. Schicchi, N., *et al.* The sub-millisievert era in CTCA: the technical basis of the new radiation dose approach. *Radiol Med* **125**, 1024-1039 (2020).
239. Wada, H., *et al.* Guideline adherence and long-term clinical outcomes in patients with acute myocardial infarction: a Japanese Registry of Acute Myocardial Infarction Diagnosed by Universal Definition (J-MINUET) substudy. *European heart journal. Acute cardiovascular care* **9**, 939-947 (2020).
240. Wu, K.C., *et al.* Associations between QT interval subcomponents, HIV serostatus, and inflammation. *Annals of noninvasive electrocardiology : the official journal of the International Society for Holter and Noninvasive Electrocardiology, Inc* **25**, e12705 (2020).
241. Abdelaziz, S.M., Hussein, R.R.S., El Mokadem, M. & Mahmoud, H.B. Clinical and hemodynamic effects of oral sildenafil on biventricular function on patients with left ventricular systolic dysfunction. *Int J Clin Pract* **75**, e14171 (2021).
242. Brust, J.C.M., *et al.* Effectiveness and Cardiac Safety of Bedaquiline-Based Therapy for Drug-Resistant Tuberculosis: A Prospective Cohort Study. *Clin Infect Dis* **73**, 2083-2092 (2021).
243. Frasch, M.G., *et al.* Brief Report: Can a Composite Heart Rate Variability Biomarker Shed New Insights About Autism Spectrum Disorder in School-Aged Children? *J Autism Dev Disord* **51**, 346-356 (2021).
244. Gasperetti, A., *et al.* Prospective use of ablation index for the ablation of right ventricle outflow tract premature ventricular contractions: a proof of concept study. *Europace : European pacing, arrhythmias, and cardiac electrophysiology : journal of the working groups on cardiac pacing, arrhythmias, and cardiac cellular electrophysiology of the European Society of Cardiology* **23**, 91-98 (2021).
245. Haji-Valizadeh, H., *et al.* Highly accelerated free-breathing real-time phase contrast cardiovascular MRI via complex-difference deep learning. *Magnetic resonance in medicine* **86**, 804-819 (2021).
246. Harding, B.N., *et al.* Longitudinal Measures of Blood Pressure and Subclinical Atrial Arrhythmias: The MESA and the ARIC Study. *Journal of the American Heart Association* **10**, e020260 (2021).
247. Hasnul, M.A., Aziz, N.A.A., Alelyani, S., Mohana, M. & Aziz, A.A. Electrocardiogram-Based Emotion Recognition Systems and Their Applications in Healthcare-A Review. *Sensors (Basel, Switzerland)* **21**(2021).
248. Hiremath, P.G., *et al.* Testosterone use and shorter electrocardiographic QT interval duration in men living with and without HIV. *HIV Med* **22**, 418-421 (2021).
249. Huang, X., Chen, P., Tang, F. & Hua, N. Detection of coronary artery disease in patients with chest pain: A machine learning model based on magnetocardiography parameters. *Clin Hemorheol Microcirc* **78**, 227-236 (2021).
250. Kacprzak, M., Brzeczek, M. & Zielinska, M. Atrial Natriuretic Peptides, Right Atrial Infarction and Prognosis of Patients with Myocardial Infarction-A Single-Center Study. *Biomolecules* **11**(2021).
251. Kwon, Y., *et al.* Pulse arrival time, a novel sleep cardiovascular marker: the multi-ethnic study of atherosclerosis. *Thorax* **76**, 1124-1130 (2021).
252. Maheshwarappa, H.M., Mishra, S., Kulkarni, A.V., Gunaseelan, V. & Kanchi, M. Use of Handheld Ultrasound Device with Artificial Intelligence for Evaluation of Cardiorespiratory System in COVID-19. *Indian J Crit Care Med* **25**, 524-527 (2021).
253. Maille, B., *et al.* Smartwatch Electrocardiogram and Artificial Intelligence for Assessing Cardiac-Rhythm Safety of Drug Therapy in the COVID-19 Pandemic. The QT-logs study. *International journal of cardiology* **331**, 333-339 (2021).
254. Martínez-Sellés, M., Juárez, M., Marina-Breysse, M., Lillo-Castellano, J.M. & Ariza, A. Rational and design of ST-segment elevation not associated with acute cardiac necrosis (LESTONNAC). A prospective registry for validation

of a deep learning system assisted by artificial intelligence. *Journal of electrocardiology* **69**, 140-144 (2021).
255. Morales, M.A., *et al.* The BrAID study protocol: integration of machine learning and transcriptomics for brugada syndrome recognition. *BMC cardiovascular disorders* **21**, 494 (2021).
256. Ogasawara, K., *et al.* Evaluation of the Potential for QTc Prolongation With Repeated Oral Doses of Fedratinib in Patients With Advanced Solid Tumors. *Clin Pharmacol Drug Dev* **10**, 366-375 (2021).
257. Pushkar, D.Y., *et al.* [Assessment of the safety and efficacy of medicinal product PPR-001 based on regulatory polypeptides of the testes]. *Urologiia*, 100-109 (2021).
258. Reinsch, N., *et al.* One-year outcome and durability of pulmonary vein isolation after prospective use of ablation index for catheter ablation in patients with persistent atrial fibrillation. *J Interv Card Electrophysiol* **62**, 143-151 (2021).
259. Riolet, C., *et al.* Clinical and prognostic implications of phenomapping in patients with heart failure receiving cardiac resynchronization therapy. *Arch Cardiovasc Dis* **114**, 197-210 (2021).
260. Roué, J.M., Morag, I., Haddad, W.M., Gholami, B. & Anand, K.J.S. Using sensor-fusion and machine-learning algorithms to assess acute pain in non-verbal infants: a study protocol. *BMJ open* **11**, e039292 (2021).
261. Santala, O.E., *et al.* Automatic Mobile Health Arrhythmia Monitoring for the Detection of Atrial Fibrillation: Prospective Feasibility, Accuracy, and User Experience Study. *JMIR mHealth and uHealth* **9**, e29933 (2021).
262. Scard, C., *et al.* Cardiac adverse events associated with anti-PD-1 therapy in patients treated for advanced melanoma: relevance of dosing troponin T levels. *Eur J Dermatol* **31**, 205-212 (2021).
263. Sun, J., *et al.* Performance evaluation of using shorter contrast injection and 70 kVp with deep learning image reconstruction for reduced contrast medium dose and radiation dose in coronary CT angiography for children: a pilot study. *Quant Imaging Med Surg* **11**, 4162-4171 (2021).
264. Xie, E., *et al.* Intermediate Markers Underlying Electrocardiographic Predictors of Incident Atrial Fibrillation: The MESA. *Circulation. Arrhythmia and electrophysiology* **14**, e009805 (2021).
265. Yoo, D.H., *et al.* Atrial Fibrillation Genomics: Discovery and Translation. *Current cardiology reports* **23**, 164 (2021).
266. Al-Zaiti, S., Macleod, R., Dam, P.V., Smith, S.W. & Birnbaum, Y. Emerging ECG methods for acute coronary syndrome detection: Recommendations & future opportunities. *Journal of electrocardiology* **74**, 65-72 (2022).
267. Bodenes, L., *et al.* Early heart rate variability evaluation enables to predict ICU patients' outcome. *Scientific reports* **12**, 2498 (2022).
268. Dilorenzo, M.P., *et al.* Ventricular Function and Tissue Characterization By Cardiac MRI in Children Following Hospitalization for Multisystem Inflammatory Syndrome in Children (MIS-C): A Prospective Study. *Res Sq* (2022).
269. Grant, L., Joo, P., Nemnom, M.J. & Thiruganasambandamoorthy, V. Machine learning versus traditional methods for the development of risk stratification scores: a case study using original Canadian Syncope Risk Score data. *Internal and emergency medicine* **17**, 1145-1153 (2022).
270. Hill, N.R., *et al.* Identification of undiagnosed atrial fibrillation using a machine learning risk prediction algorithm and diagnostic testing (PULsE-AI) in primary care: cost-effectiveness of a screening strategy evaluated in a randomized controlled trial in England. *J Med Econ* **25**, 974-983 (2022).
271. Hill, N.R., *et al.* Identification of undiagnosed atrial fibrillation using a machine learning risk-prediction algorithm and diagnostic testing (PULsE-AI) in primary care: a multi-centre randomized controlled trial in England. *European heart journal. Digital health* **3**, 195-204 (2022).
272. Kim, S., *et al.* System of integrating biosignals during hemodialysis: the CONTINUAL (Continuous mOnitoriNg viTal sIgN dUring hemodiALysis) registry. *Kidney Res Clin Pract* **41**, 363-371 (2022).

273. Ng, B., Nayyar, S. & Chauhan, V.S. The Role of Artificial Intelligence and Machine Learning in Clinical Cardiac Electrophysiology. *The Canadian journal of cardiology* **38**, 246-258 (2022).
274. Steger, A., *et al.* The Polyscore of autonomic parameters predicts mortality and identifies low-risk individuals among diabetic survivors of acute myocardial infarction. *Scientific reports* **12**, 6069 (2022).
275. Sun, Y., *et al.* Contactless facial video recording with deep learning models for the detection of atrial fibrillation. *Scientific reports* **12**, 281 (2022).
276. Unal, A., Arsava, E.M., Caglar, G. & Topcuoglu, M.A. Alarms in a neurocritical care unit: a prospective study. *J Clin Monit Comput* **36**, 995-1001 (2022).
277. van Beek, S.W., *et al.* Model-Predicted Impact of ECG Monitoring Strategies During Bedaquiline Treatment. *Open Forum Infect Dis* **9**, ofac372 (2022).
278. Vos, W., *et al.* The 2000HIV study: Design, multi-omics methods and participant characteristics. *Front Immunol* **13**, 982746 (2022).
279. Bjerkén, L.V., Rønborg, S.N., Jensen, M.T., Ørting, S.N. & Nielsen, O.W. Artificial intelligence enabled ECG screening for left ventricular systolic dysfunction: a systematic review. *Heart Fail Rev* **28**, 419-430 (2023).
280. Bona, R., Marini, P., Turilli, D., Masala, S. & Scaglione, M. Coronary Computed Tomography Angiography with Deep Learning Image Reconstruction: A Preliminary Study to Evaluate Radiation Exposure Reduction. *Tomography* **9**, 1019-1028 (2023).
281. Canning, C., Guo, J., Narang, A., Thomas, J.D. & Ahmad, F.S. The Emerging Role of Artificial Intelligence in Valvular Heart Disease. *Heart Fail Clin* **19**, 391-405 (2023).
282. Danilov, A. & Aronow, W.S. Artificial Intelligence in Cardiology: Applications and Obstacles. *Current problems in cardiology* **48**, 101750 (2023).
283. de, A.F.F., *et al.* A machine learning method integrating ECG and gated SPECT for cardiac resynchronization therapy decision support. *Eur J Nucl Med Mol Imaging* **50**, 3022-3033 (2023).
284. Di Costanzo, A., Indolfi, C., Franzone, A., Esposito, G. & Spaccarotella, C.A.M. Lp(a) in the Pathogenesis of Aortic Stenosis and Approach to Therapy with Antisense Oligonucleotides or Short Interfering RNA. *Int J Mol Sci* **24**(2023).
285. DiLorenzo, M.P., *et al.* Ventricular function and tissue characterization by cardiac magnetic resonance imaging following hospitalization for multisystem inflammatory syndrome in children: a prospective study. *Pediatr Radiol* **53**, 394-403 (2023).
286. ElRefai, M., *et al.* Role of deep learning methods in screening for subcutaneous implantable cardioverter defibrillator in heart failure. *Annals of noninvasive electrocardiology : the official journal of the International Society for Holter and Noninvasive Electrocardiology, Inc* **28**, e13028 (2023).
287. Jin, Y., *et al.* The effect of anti-tuberculosis drug pharmacokinetics on QTc prolongation. *Int J Antimicrob Agents* **62**, 106939 (2023).
288. Kędzierski, B., *et al.* Radiation Doses in Cardiovascular Computed Tomography. *Life (Basel)* **13**(2023).
289. Morales, M.A., *et al.* Highly accelerated free-breathing real-time myocardial tagging for exercise cardiovascular magnetic resonance. *J Cardiovasc Magn Reson* **25**, 56 (2023).
290. Neglia, D., *et al.* Use of cardiac imaging in chronic coronary syndromes: the EURECA Imaging registry. *European heart journal* **44**, 142-158 (2023).
291. Rahim, F.O., *et al.* Longitudinal ECG changes among adults with HIV in Tanzania: A prospective cohort study. *PLOS Glob Public Health* **3**, e0002525 (2023).

292. Ranjbar, A., Ravn, J., Ronningen, E. & Hanseth, O. Enabling Clinical Trials of Artificial Intelligence: Infrastructure for Heart Failure Predictions. *Studies in health technology and informatics* **302**, 177-181 (2023).
293. Rinkel, L.A.*, et al.* Cardiac thrombi detected by CT in patients with acute ischemic stroke: A substudy of Mind the Heart. *Eur Stroke J* **8**, 168-174 (2023).
294. Rinkel, L.A.*, et al.* Detection of patent foramen ovale in patients with ischemic stroke on prospective ECG-gated cardiac CT compared to transthoracic echocardiography. *J Neurol* **270**, 3537-3542 (2023).
295. Sabatino, F.*, et al.* Extensive Posterior Wall Isolation on Top of Pulmonary Vein Isolation Guided by Ablation Index in Persistent Atrial Fibrillation Ablation. *Life (Basel)* **13**(2023).
296. Sakita, F.M.*, et al.* Six-month blood pressure and glucose control among HIV-infected adults with elevated blood pressure and hyperglycemia in northern Tanzania: A prospective observational study. *PloS one* **18**, e0285472 (2023).
297. Selder, J.L.*, et al.* Accuracy of a Standalone Atrial Fibrillation Detection Algorithm Added to a Popular Wristband and Smartwatch: Prospective Diagnostic Accuracy Study. *Journal of medical Internet research* **25**, e44642 (2023).
298. Six, S.*, et al.* Patient-reported outcome measures on mental health and psychosocial factors in patients with Brugada syndrome. *Europace : European pacing, arrhythmias, and cardiac electrophysiology : journal of the working groups on cardiac pacing, arrhythmias, and cardiac cellular electrophysiology of the European Society of Cardiology* **25**(2023).
299. Stark, K.*, et al.* Six month incidence of major adverse cardiovascular events among adults with HIV in northern Tanzania: a prospective observational study. *BMJ open* **13**, e075275 (2023).
300. Thaweethai, T.*, et al.* Development of a Definition of Postacute Sequelae of SARS-CoV-2 Infection. *Jama* **329**, 1934-1946 (2023).
301. Yang, F.*, et al.* Effect of 320-Row Computed Tomography Acquisition Technology on Coronary Computed Tomography Angiography-Derived Fractional Flow Reserve Based on Machine Learning: Systolic and Diastolic Scan Acquisition. *J Comput Assist Tomogr* **47**, 205-211 (2023).
302. Allescher, J.*, et al.* QRS fragmentation does not predict mortality in survivors of acute myocardial infarction. *Clin Cardiol* **47**, e24218 (2024).
303. Arsh, H.*, et al.* Electrocardiographic changes in pneumothorax: an updated review. *Ann Med Surg (Lond)* **86**, 3551-3556 (2024).
304. Boehmer, A.A.*, et al.* Angiotensin Receptor-Neprilysin Inhibitor Is Associated With Improved Cardiac Autonomic Function in Heart Failure. *Journal of the American Heart Association* **13**, e033538 (2024).
305. Brandberg, H., Sundberg, C.J., Spaak, J., Koch, S. & Kahan, T. Are medical history data fit for risk stratification of patients with chest pain in emergency care? Comparing data collected from patients using computerized history taking with data documented by physicians in the electronic health record in the CLEOS-CPDS prospective cohort study. *Journal of the American Medical Informatics Association : JAMIA* **31**, 1529-1539 (2024).
306. Caronna, E.*, et al.* Redefining migraine prevention: early treatment with anti-CGRP monoclonal antibodies enhances response in the real world. *J Neurol Neurosurg Psychiatry* **95**, 927-937 (2024).
307. Choi, S.H. Spiking neural networks for biomedical signal analysis. *Biomed Eng Lett* **14**, 955-966 (2024).
308. D'Amario, D.*, et al.* Ticagrelor enhances the cardioprotective effects of ischemic preconditioning in stable patients undergoing percutaneous coronary intervention: the TAPER-S randomized study. *Eur Heart J Cardiovasc Pharmacother* **10**, 190-200 (2024).
309. Handra, J.*, et al.* The Role of Machine Learning in the Detection of Cardiac Fibrosis in Electrocardiograms:

Scoping Review. *JMIR Cardio* **8**, e60697 (2024).

310. Hulot, J.S., *et al.* Rationale and design of the PACIFIC-PRESERVED (PhenomApping, ClassIFication and Innovation for Cardiac dysfunction in patients with heart failure and PRESERVED left ventricular ejection fraction) study. *Arch Cardiovasc Dis* **117**, 332-342 (2024).

311. Islam, M.S., *et al.* Diagnostic and Prognostic Electrocardiogram-Based Models for Rapid Clinical Applications. *The Canadian journal of cardiology* **40**, 1788-1803 (2024).

312. Janik, M., Raad, G., Nijmeh, G., O'Steen, M. & Rasmussen, J. Diagnostic accuracy for detecting atrial fibrillation using a novel machine learning algorithm in a blood pressure monitor. *Heart Rhythm* **21**, 2023-2027 (2024).

313. Jia, Y. & Leung, S.W. The efficacy of Chinese herbal drugs for adults with angina pectoris: Bayesian network meta-analysis of 331 RCTs involving 36,467 individuals. *J Ethnopharmacol* **326**, 117925 (2024).

314. Joy, G., *et al.* Electrophysiological Characterization of Subclinical and Overt Hypertrophic Cardiomyopathy by Magnetic Resonance Imaging-Guided Electrocardiography. *Journal of the American College of Cardiology* **83**, 1042-1055 (2024).

315. Lampert, R., *et al.* Vigorous Exercise in Patients With Congenital Long QT Syndrome: Results of the Prospective, Observational, Multinational LIVE-LQTS Study. *Circulation* **150**, 516-530 (2024).

316. Laubham, M., *et al.* Patient Driven EKG Device Performance in Adults with Fontan Palliation. *Pediatr Cardiol* (2024).

317. May, A.M. & Kashou, A.H. A novel way to prospectively evaluate of AI-enhanced ECG algorithms. *Journal of electrocardiology* **86**, 153756 (2024).

318. Ryu, S., Yun, S., Lee, S. & Jeong, I.C. Exploring the Possibility of Photoplethysmography-Based Human Activity Recognition Using Convolutional Neural Networks. *Sensors (Basel, Switzerland)* **24**(2024).

319. Schäfer, A.C., *et al.* Effects of baroreflex activation therapy on cardiac function and morphology. *ESC Heart Fail* **11**, 3360-3367 (2024).

320. Toprak, B., *et al.* Diagnostic accuracy of a machine learning algorithm using point-of-care high-sensitivity cardiac troponin I for rapid rule-out of myocardial infarction: a retrospective study. *The Lancet. Digital health* **6**, e729-e738 (2024).

321. Widmann, M., *et al.* COVID-19 in Female and Male Athletes: Symptoms, Clinical Findings, Outcome, and Prolonged Exercise Intolerance-A Prospective, Observational, Multicenter Cohort Study (CoSmo-S). *Sports Med* **54**, 1033-1049 (2024).

322. Yuan, D., *et al.* Evaluation of image quality on low contrast media with deep learning image reconstruction algorithm in prospective ECG-triggering coronary CT angiography. *Int J Cardiovasc Imaging* **40**, 1377-1388 (2024).

323. Ahluwalia, N., *et al.* The Restitution Threshold Index Characterizes the Association Between Atrial Fibrillation Ventricular Rate and Ejection Fraction. *JACC. Clinical electrophysiology* **11**, 282-294 (2025).

324. Anisuzzaman, D.M., *et al.* Leveraging Comprehensive Echo Data to Power Artificial Intelligence Models for Handheld Cardiac Ultrasound. *Mayo Clin Proc Digit Health* **3**, 100194 (2025).

325. Aziz-Safaie, T., *et al.* Fast and Robust Single-Shot Cine Cardiac MRI Using Deep Learning Super-Resolution Reconstruction. *Invest Radiol* (2025).

326. Biswas, D., *et al.* Transforming Population Health Screening for Atherosclerotic Cardiovascular Disease with AI-Enhanced ECG Analytics: Opportunities and Challenges. *Curr Atheroscler Rep* **27**, 86 (2025).

327. Boettger, P., *et al.* Short-duration atrial fibrillation in ischemic stroke: high risk despite subclinical burden-a

prospective cohort study. *BMC cardiovascular disorders* **25**, 616 (2025).
328. Cagnina, A., *et al.* Assessing the need for coronary angiography in high-risk non-ST-elevation acute coronary syndrome patients using artificial intelligence and computed tomography. *Int J Cardiovasc Imaging* **41**, 55-61 (2025).
329. Cramer, I., *et al.* Accuracy of remote, video-based supraventricular tachycardia detection in patients undergoing elective electrical cardioversion: a prospective cohort. *J Clin Monit Comput* (2025).
330. Fahrni, G., *et al.* Trading off Iodine and Radiation Dose in Coronary Computed Tomography. *J Cardiovasc Dev Dis* **12**(2025).
331. Hamelink, I., van Tuinen, M., Kwee, T.C., van Ooijen, P.M.A. & Vliegenthart, R. Repeatability of AI-based, automatic measurement of vertebral and cardiovascular imaging biomarkers in low-dose chest CT: the ImaLife cohort. *European radiology* **35**, 3833-3841 (2025).
332. Haneda, E., *et al.* Bolus tracking from pulsed x-ray projections: A feasibility study using a five-dimensional cardiac CT contrast dynamics model. *Medical physics* **52**, 131-145 (2025).
333. Hannane, N., *et al.* Long-term prediction of mortality by heart rate turbulence in hemodialysis patients and the impact of diabetes mellitus-a longitudinal observational study. *J Nephrol* (2025).
334. Hassan, N., *et al.* Electrocardiographic abnormalities in epilepsy: analysis of cardiac conduction patterns and SUDEP Risk. *Neurol Sci* (2025).
335. Heseltine-Carp, W., *et al.* Machine learning to predict stroke risk from routine hospital data: A systematic review. *International journal of medical informatics* **196**, 105811 (2025).
336. Jayagopal, P.B., *et al.* Do gender differences matter in Acute Heart Failure? Insights from Indian College of Cardiology - National Heart Failure Registry, India. *Int J Cardiol Cardiovasc Risk Prev* **26**, 200441 (2025).
337. Jha, C.K. Automated cardiac arrhythmia detection techniques: a comprehensive review for prospective approach. *Comput Methods Biomech Biomed Engin* **28**, 1639-1654 (2025).
338. Khan, S., *et al.* Efficacy of AI Models in Detecting Heart Failure Using ECG Data: A Systematic Review and Meta-Analysis. *Cureus* **17**, e78683 (2025).
339. Kim, A., *et al.* Artificial intelligence for electrocardiographic diagnosis of perioperative myocardial ischaemia: a scoping review. *Br J Anaesth* **135**, 561-570 (2025).
340. Kim, S. Predicting Chemotherapy-Induced Peripheral Neuropathy Using Transformer-Based Multimodal Deep Learning. *Research (Wash D C)* **8**, 0795 (2025).
341. Klein, L., *et al.* Noninvasive Pulmonary Capillary Wedge Pressure Estimation in Heart Failure Patients With the Use of Wearable Sensing and AI. *JACC Heart Fail* **13**, 102513 (2025).
342. Li, Y., *et al.* The autonomic response following taVNS predicts changes in level of consciousness in DoC patients. *Scientific reports* **15**, 7317 (2025).
343. Malin, E.J., *et al.* Library of realistic 4D digital beating heart models based on patient CT data. *Medical physics* **52**, e17945 (2025).
344. Malins, J.G., *et al.* Snapshot artificial intelligence-determination of ejection fraction from a single frame still image: a multi-institutional, retrospective model development and validation study. *The Lancet. Digital health* **7**, e255-e263 (2025).
345. Masini, G., *et al.* Markers of Left Atrial Myopathy: Prognostic Usefulness for Ischemic Stroke and Dementia in People in Sinus Rhythm. *Stroke* **56**, 858-867 (2025).
346. Mohyeldin, M., *et al.* Artificial Intelligence in Hypertrophic Cardiomyopathy: Advances, Challenges, and Future

Directions for Personalized Risk Prediction and Management. *Cureus* **17**, e87907 (2025).
347. Nguyen, K.T., *et al.* Racial and Ethnic Representation and Study Engagement in a Siteless Digital Clinical Trial Using a Smartwatch: Findings From the Apple Heart Study. *Mayo Clin Proc Digit Health* **3**, 100232 (2025).
348. Popat, A., *et al.* The Efficacy of Artificial Intelligence in the Detection and Management of Atrial Fibrillation. *Cureus* **17**, e77135 (2025).
349. Shiri, I., *et al.* Multi-modality artificial intelligence-based transthyretin amyloid cardiomyopathy detection in patients with severe aortic stenosis. *Eur J Nucl Med Mol Imaging* **52**, 485-500 (2025).
350. Tran, H.H., *et al.* Electrocardiogram-Based Artificial Intelligence for Detection of Low Ejection Fraction: A Contemporary Review. *Cardiol Rev* (2025).
351. Tran, H.H., *et al.* AI-Guided Decision Support in Acute Cardiac Care: From Chest Pain to STEMI. *Cardiol Rev* (2025).
352. Tremamunno, G., *et al.* Comparative analysis of pre-transcatheter aortic valve implantation CTA protocols: Optimizing radiation dose and contrast volume. *International journal of cardiology* **437**, 133514 (2025).
353. Kennedy, R.L., *et al.* An artificial neural network system for diagnosis of acute myocardial infarction (AMI) in the accident and emergency department: evaluation and comparison with serum myoglobin measurements. *Computer methods and programs in biomedicine* **52**, 93-103 (1997).
354. Liu, N., *et al.* An intelligent scoring system and its application to cardiac arrest prediction. *IEEE Trans Inf Technol Biomed* **16**, 1324-1331 (2012).
355. Ong, M.E., *et al.* Prediction of cardiac arrest in critically ill patients presenting to the emergency department using a machine learning score incorporating heart rate variability compared with the modified early warning score. *Critical care (London, England)* **16**, R108 (2012).
356. Nemati, S., *et al.* An Interpretable Machine Learning Model for Accurate Prediction of Sepsis in the ICU. *Critical care medicine* **46**, 547-553 (2018).
357. Galloway, C.D., *et al.* Development and Validation of a Deep-Learning Model to Screen for Hyperkalemia From the Electrocardiogram. *JAMA cardiology* **4**, 428-436 (2019).
358. Al-Zaiti, S., *et al.* Machine learning-based prediction of acute coronary syndrome using only the pre-hospital 12-lead electrocardiogram. *Nature communications* **11**, 3966 (2020).
359. Di Lorenzo Oliveira, C., *et al.* Risk Score for Predicting 2-Year Mortality in Patients With Chagas Cardiomyopathy From Endemic Areas: SaMi-Trop Cohort Study. *Journal of the American Heart Association* **9**, e014176 (2020).
360. Jing, L., *et al.* A Machine Learning Approach to Management of Heart Failure Populations. *JACC Heart Fail* **8**, 578-587 (2020).
361. Bouzid, Z., *et al.* In Search of an Optimal Subset of ECG Features to Augment the Diagnosis of Acute Coronary Syndrome at the Emergency Department. *Journal of the American Heart Association* **10**, e017871 (2021).
362. Bouzid, Z., *et al.* Novel ECG features and machine learning to optimize culprit lesion detection in patients with suspected acute coronary syndrome. *Journal of electrocardiology* **69s**, 31-37 (2021).
363. Khurshid, S., *et al.* Deep Learning to Predict Cardiac Magnetic Resonance-Derived Left Ventricular Mass and Hypertrophy From 12-Lead ECGs. *Circ Cardiovasc Imaging* **14**, e012281 (2021).
364. Unterhuber, M., *et al.* Deep learning detects heart failure with preserved ejection fraction using a baseline electrocardiogram. *European heart journal. Digital health* **2**, 699-703 (2021).
365. Siva Kumar, S., *et al.* Machine learning derived ECG risk score improves cardiovascular risk assessment in conjunction with coronary artery calcium scoring. *Frontiers in cardiovascular medicine* **9**, 976769 (2022).

366. Suzuki, S., *et al.* Identifying patients with atrial fibrillation during sinus rhythm on ECG: Significance of the labeling in the artificial intelligence algorithm. *Int J Cardiol Heart Vasc* **38**, 100954 (2022).
367. Adedinsewo, D., *et al.* Non-invasive detection of cardiac allograft rejection among heart transplant recipients using an electrocardiogram based deep learning model. *European heart journal. Digital health* **4**, 71-80 (2023).
368. de Capretz, P.O., *et al.* Machine learning for early prediction of acute myocardial infarction or death in acute chest pain patients using electrocardiogram and blood tests at presentation. *BMC medical informatics and decision making* **23**, 25 (2023).
369. Fan, Z., *et al.* Comparing the performance of machine learning and conventional models for predicting atherosclerotic cardiovascular disease in a general Chinese population. *BMC medical informatics and decision making* **23**, 134 (2023).
370. Hirota, N., *et al.* Identification of patients with dilated phase of hypertrophic cardiomyopathy using a convolutional neural network applied to multiple, dual, and single lead electrocardiograms. *Int J Cardiol Heart Vasc* **46**, 101211 (2023).
371. Liu, Z., *et al.* Accurate detection of arrhythmias on raw electrocardiogram images: An aggregation attention multi-label model for diagnostic assistance. *Med Eng Phys* **114**, 103964 (2023).
372. Sangha, V., *et al.* Detection of Left Ventricular Systolic Dysfunction From Electrocardiographic Images. *Circulation* **148**, 765-777 (2023).
373. Shiraishi, Y., *et al.* Improved prediction of sudden cardiac death in patients with heart failure through digital processing of electrocardiography. *Europace : European pacing, arrhythmias, and cardiac electrophysiology : journal of the working groups on cardiac pacing, arrhythmias, and cardiac cellular electrophysiology of the European Society of Cardiology* **25**, 922-930 (2023).
374. Arita, T., *et al.* Identifying patients with acute aortic dissection using an electrocardiogram with convolutional neural network. *Int J Cardiol Heart Vasc* **51**, 101389 (2024).
375. Binsawad, M. Enhancing kidney disease prediction with optimized forest and ECG signals data. *Heliyon* **10**, e30792 (2024).
376. Cho, Y., *et al.* Artificial Intelligence-Based Electrocardiographic Biomarker for Outcome Prediction in Patients With Acute Heart Failure: Prospective Cohort Study. *Journal of medical Internet research* **26**, e52139 (2024).
377. Dhingra, L.S., *et al.* Artificial Intelligence Enabled Prediction of Heart Failure Risk from Single-lead Electrocardiograms. *medRxiv* (2024).
378. Dhingra, L.S., *et al.* An Ensemble Deep Learning Algorithm for Structural Heart Disease Screening Using Electrocardiographic Images: PRESENT SHD. *medRxiv* (2024).
379. Duong, S.Q., *et al.* Quantitative Prediction of Right Ventricular Size and Function From the ECG. *Journal of the American Heart Association* **13**, e031671 (2024).
380. Gilbers, M.D., *et al.* Clinical Predictors of Device-Detected Atrial Fibrillation During 2.5 Years After Cardiac Surgery: Prospective RACE V Cohort. *JACC. Clinical electrophysiology* **10**, 941-955 (2024).
381. Jeong, H., *et al.* Prediction of intraoperative hypotension using deep learning models based on non-invasive monitoring devices. *J Clin Monit Comput* **38**, 1357-1365 (2024).
382. Jeong, J.H., *et al.* Deep learning algorithm for predicting left ventricular systolic dysfunction in atrial fibrillation with rapid ventricular response. *European heart journal. Digital health* **5**, 683-691 (2024).
383. Kallonen, A., *et al.* Early detection of late-onset neonatal sepsis from noninvasive biosignals using deep learning: A multicenter prospective development and validation study. *International journal of medical informatics* **184**,

105366 (2024).
384. Kanaji, Y., *et al.* Predictive Value of Artificial Intelligence-Enabled Electrocardiography in Patients With Takotsubo Cardiomyopathy. *Journal of the American Heart Association* **13**, e031859 (2024).
385. Kany, S., *et al.* Integrating Clinical, Genetic, and Electrocardiogram-Based Artificial Intelligence to Estimate Risk of Incident Atrial Fibrillation. *medRxiv* (2024).
386. König, S., *et al.* Artificial intelligence-based identification of left ventricular systolic dysfunction from 12-lead electrocardiograms: external validation and advanced application of an existing model. *European heart journal. Digital health* **5**, 144-151 (2024).
387. Lee, S.H., *et al.* Development of Clinically Validated Artificial Intelligence Model for Detecting ST-segment Elevation Myocardial Infarction. *Annals of emergency medicine* **84**, 540-548 (2024).
388. Peng, X., *et al.* A multimodal physiological and psychological dataset for human with mental stress induced myocardial ischemia. *Scientific data* **11**, 704 (2024).
389. Pham, H.N., *et al.* Dynamic electrocardiogram changes are a novel risk marker for sudden cardiac death. *European heart journal* **45**, 809-819 (2024).
390. Siontis, K.C., *et al.* Hypertrophic cardiomyopathy detection with artificial intelligence electrocardiography in international cohorts: an external validation study. *European heart journal. Digital health* **5**, 416-426 (2024).
391. Suzuki, S., *et al.* Lead-Specific Performance for Atrial Fibrillation Detection in Convolutional Neural Network Models Using Sinus Rhythm Electrocardiography. *Circ Rep* **6**, 46-54 (2024).
392. van de Leur, R.R., *et al.* Automatic triage of twelve-lead electrocardiograms using deep convolutional neural networks: a first implementation study. *European heart journal. Digital health* **5**, 89-96 (2024).
393. Weidlich, S., *et al.* Reducing the burden of inconclusive smart device single-lead ECG tracings via a novel artificial intelligence algorithm. *Cardiovasc Digit Health J* **5**, 29-35 (2024).
394. Adel, F.W., *et al.* Artificial intelligence evaluation of electrocardiographic characteristics and interval changes in transgender patients on gender-affirming hormone therapy. *European heart journal. Digital health* **6**, 55-62 (2025).
395. Ahn, J.C., *et al.* AI-Cirrhosis-ECG (ACE) score for predicting decompensation and liver outcomes. *JHEP Rep* **7**, 101356 (2025).
396. Baker, P.O., *et al.* Artificial Intelligence Driven Prehospital ECG Interpretation for the Reduction of False Positive Emergent Cardiac Catheterization Lab Activations: A Retrospective Cohort Study. *Prehospital emergency care : official journal of the National Association of EMS Physicians and the National Association of State EMS Directors* **29**, 218-226 (2025).
397. Bouzid, Z., *et al.* Electrocardiogram-based machine learning for risk stratification of patients with suspected acute coronary syndrome. *European heart journal* **46**, 943-954 (2025).
398. Brandberg, H., *et al.* Performance of computerized self-reported medical history taking and HEAR score for safe early rule-out of cardiac events in acute chest pain patients: the CLEOS-CPDS prospective cohort study. *European heart journal. Digital health* **6**, 104-114 (2025).
399. Chang, P.C., *et al.* Utilizing 12-lead electrocardiogram and machine learning to retrospectively estimate and prospectively predict atrial fibrillation and stroke risk. *Comput Biol Med* **188**, 109871 (2025).
400. Choi, J.W.H., *et al.* AI-enhanced recognition of occlusions in acute coronary syndrome (AERO-ACS): a retrospective study. *Coron Artery Dis* (2025).
401. Croon, P.M., Dhingra, L.S., Biswas, D., Oikonomou, E.K. & Khera, R. Phenotypic Selectivity of Artificial Intelligence-enhanced Electrocardiography in Cardiovascular Diagnosis and Risk Prediction. *Circulation* (2025).

402. Desai, M.Y., *et al.* Real-World Artificial Intelligence-Based Electrocardiographic Analysis to Diagnose Hypertrophic Cardiomyopathy. *JACC. Clinical electrophysiology* **11**, 1324-1333 (2025).
403. Dhingra, L.S., *et al.* Artificial Intelligence-Enabled Prediction of Heart Failure Risk From Single-Lead Electrocardiograms. *JAMA cardiology* **10**, 574-584 (2025).
404. Dhingra, L.S., *et al.* Ensemble Deep Learning Algorithm for Structural Heart Disease Screening Using Electrocardiographic Images: PRESENT SHD. *Journal of the American College of Cardiology* **85**, 1302-1313 (2025).
405. Kalousios, S., *et al.* ECG-based epileptic seizure prediction: Challenges of current data-driven models. *Epilepsia Open* **10**, 143-154 (2025).
406. Khunte, A., *et al.* Artificial Intelligence-Based Automated Interpretation of Images of Electrocardiograms: Development and Multinational Validation of ECG-GPT. *medRxiv* (2025).
407. Mayourian, J., Geggel, R., La Cava, W.G., Ghelani, S.J. & Triedman, J.K. Pediatric Electrocardiogram-Based Deep Learning to Predict Secundum Atrial Septal Defects. *Pediatr Cardiol* **46**, 1235-1240 (2025).
408. Pan, Y., *et al.* Continuous atrial fibrillation monitoring using a wearable smartwatch: Using long-term Holter as reference. *Digital health* **11**, 20552076251314105 (2025).
409. Poterucha, T.J., *et al.* Detecting structural heart disease from electrocardiograms using AI. *Nature* **644**, 221-230 (2025).
410. Sakuma, M., *et al.* Utility of convolutional neural network-enhanced electrocardiogram to diagnose and predict mitral regurgitation in patients with chronic atrial fibrillation. *Heart and vessels* (2025).
411. Schlesinger, D.E., *et al.* Artificial intelligence for hemodynamic monitoring with a wearable electrocardiogram monitor. *Commun Med (Lond)* **5**, 4 (2025).
412. Schoels, M., *et al.* Artificial intelligence for prediction of atrial fibrillation in the stroke unit: a retrospective derivation validation cohort study. *EBioMedicine* **118**, 105869 (2025).
413. Singh, M., *et al.* Role of Biological Age in the Determination of Long-Term Cause-Specific Death Following Percutaneous Coronary Interventions. *Journal of the American Heart Association* **14**, e036876 (2025).
414. Trivedi, R.K., Chiu, I.M., Hughes, J.W., Rogers, A.J. & Ouyang, D. Deep learning on electrocardiogram waveforms to stratify risk of obstructive stable coronary artery disease. *European heart journal. Digital health* **6**, 456-465 (2025).
415. Wang, L., Wu, H., Wu, C., Shu, L. & Zhou, D. A deep-learning system integrating electrocardiograms and laboratory indicators for diagnosing acute aortic dissection and acute myocardial infarction. *International journal of cardiology* **423**, 133008 (2025).
416. Yu, C.C., *et al.* ECG-based machine learning model for AF identification in patients with first ischemic stroke. *Int J Stroke* **20**, 411-418 (2025).
417. Baxt, W.G. & Skora, J. Prospective validation of artificial neural network trained to identify acute myocardial infarction. *Lancet (London, England)* **347**, 12-15 (1996).
418. Baxt, W.G., Shofer, F.S., Sites, F.D. & Hollander, J.E. A neural computational aid to the diagnosis of acute myocardial infarction. *Annals of emergency medicine* **39**, 366-373 (2002).
419. Harrison, R.F. & Kennedy, R.L. Artificial neural network models for prediction of acute coronary syndromes using clinical data from the time of presentation. *Annals of emergency medicine* **46**, 431-439 (2005).
420. Forberg, J.L., *et al.* An artificial neural network to safely reduce the number of ambulance ECGs transmitted for physician assessment in a system with prehospital detection of ST elevation myocardial infarction. *Scandinavian*

journal of trauma, resuscitation and emergency medicine **20**, 8 (2012).
421. Melillo, P., Orrico, A., Scala, P., Crispino, F. & Pecchia, L. Cloud-Based Smart Health Monitoring System for Automatic Cardiovascular and Fall Risk Assessment in Hypertensive Patients. *Journal of medical systems* **39**, 109 (2015).
422. Attia, Z.I.*, et al.* Prospective validation of a deep learning electrocardiogram algorithm for the detection of left ventricular systolic dysfunction. *Journal of cardiovascular electrophysiology* **30**, 668-674 (2019).
423. Kagiyama, N.*, et al.* Machine Learning Assessment of Left Ventricular Diastolic Function Based on Electrocardiographic Features. *Journal of the American College of Cardiology* **76**, 930-941 (2020).
424. Fu, W. & Li, R. Diagnostic performance of a wearing dynamic ECG recorder for atrial fibrillation screening: the HUAMI heart study. *BMC cardiovascular disorders* **21**, 558 (2021).
425. Giudicessi, J.R.*, et al.* Artificial Intelligence-Enabled Assessment of the Heart Rate Corrected QT Interval Using a Mobile Electrocardiogram Device. *Circulation* **143**, 1274-1286 (2021).
426. Kasaeyan Naeini, E.*, et al.* Pain Recognition With Electrocardiographic Features in Postoperative Patients: Method Validation Study. *Journal of medical Internet research* **23**, e25079 (2021).
427. Lin, C.*, et al.* Artificial Intelligence-Assisted Electrocardiography for Early Diagnosis of Thyrotoxic Periodic Paralysis. *J Endocr Soc* **5**, bvab120 (2021).
428. Attia, Z.I.*, et al.* Automated detection of low ejection fraction from a one-lead electrocardiogram: application of an AI algorithm to an electrocardiogram-enabled Digital Stethoscope(). *European heart journal. Digital health* **3**, 373-379 (2022).
429. Attia, Z.I.*, et al.* Prospective evaluation of smartwatch-enabled detection of left ventricular dysfunction. *Nature medicine* **28**, 2497-2503 (2022).
430. Bachtiger, P.*, et al.* Point-of-care screening for heart failure with reduced ejection fraction using artificial intelligence during ECG-enabled stethoscope examination in London, UK: a prospective, observational, multicentre study. *The Lancet. Digital health* **4**, e117-e125 (2022).
431. Chokshi, S.*, et al.* Comparison Between QT and Corrected QT Interval Assessment by an Apple Watch With the AccurBeat Platform and by a 12-Lead Electrocardiogram With Manual Annotation: Prospective Observational Study. *JMIR Form Res* **6**, e41241 (2022).
432. Liu, W.T.*, et al.* A Deep-Learning Algorithm-Enhanced System Integrating Electrocardiograms and Chest X-rays for Diagnosing Aortic Dissection. *The Canadian journal of cardiology* **38**, 160-168 (2022).
433. Mannhart, D.*, et al.* Clinical Validation of Automated Corrected QT-Interval Measurements From a Single Lead Electrocardiogram Using a Novel Smartwatch. *Frontiers in cardiovascular medicine* **9**, 906079 (2022).
434. Ng, A.*, et al.* Predicting the Next-Day Perceived and Physiological Stress of Pregnant Women by Using Machine Learning and Explainability: Algorithm Development and Validation. *JMIR mHealth and uHealth* **10**, e33850 (2022).
435. Patel, H.B.*, et al.* Electrocardiogram-Based Machine Learning Emulator Model for Predicting Novel Echocardiography-Derived Phenogroups for Cardiac Risk-Stratification: A Prospective Multicenter Cohort Study. *J Patient Cent Res Rev* **9**, 98-107 (2022).
436. Quartieri, F.*, et al.* Artificial intelligence augments detection accuracy of cardiac insertable cardiac monitors: Results from a pilot prospective observational study. *Cardiovasc Digit Health J* **3**, 201-211 (2022).
437. Yang, Y.*, et al.* A Multimodal AI System for Out-of-Distribution Generalization of Seizure Identification. *IEEE J Biomed Health Inform* **26**, 3529-3538 (2022).

438. Adedinsewo, D., *et al.* RApid Throughput Screening for Asymptomatic COVID-19 Infection With an Electrocardiogram: A Prospective Observational Study. *Mayo Clin Proc Digit Health* **1**, 455-466 (2023).
439. Bouzid, Z., *et al.* Incorporation of Serial 12-Lead Electrocardiogram With Machine Learning to Augment the Out-of-Hospital Diagnosis of Non-ST Elevation Acute Coronary Syndrome. *Annals of emergency medicine* **81**, 57-69 (2023).
440. Hennings, E., *et al.* Assessment of the atrial fibrillation burden in Holter electrocardiogram recordings using artificial intelligence. *Cardiovasc Digit Health J* **4**, 41-47 (2023).
441. Poh, M.Z., *et al.* Validation of a Deep Learning Algorithm for Continuous, Real-Time Detection of Atrial Fibrillation Using a Wrist-Worn Device in an Ambulatory Environment. *Journal of the American Heart Association* **12**, e030543 (2023).
442. Surendra, K., *et al.* Pragmatic screening for heart failure in the general population using an electrocardiogram-based neural network. *ESC Heart Fail* **10**, 975-984 (2023).
443. Adedinsewo, D., *et al.* Artificial intelligence-based screening for cardiomyopathy in an obstetric population: A pilot study. *Cardiovasc Digit Health J* **5**, 132-140 (2024).
444. Chiu, I.M., *et al.* Serum Potassium Monitoring Using AI-Enabled Smartwatch Electrocardiograms. *JACC. Clinical electrophysiology* **10**, 2644-2654 (2024).
445. Ding, T.Y., *et al.* Epileptic seizure forecasting with wearable-based nocturnal sleep features. *Epilepsia Open* **9**, 1793-1805 (2024).
446. Gharbi, O., *et al.* Detection of focal to bilateral tonic-clonic seizures using a connected shirt. *Epilepsia* **65**, 2280-2294 (2024).
447. Liu, W.T., *et al.* Opportunistic Screening for Asymptomatic Left Ventricular Dysfunction With the Use of Electrocardiographic Artificial Intelligence: A Cost-Effectiveness Approach. *The Canadian journal of cardiology* **40**, 1310-1321 (2024).
448. Munoz-Macho, A.A., Dominguez-Morales, M.J. & Sevillano-Ramos, J.L. Analyzing ECG signals in professional football players using machine learning techniques. *Heliyon* **10**, e26789 (2024).
449. Altinbilek, E., *et al.* Task-specific versus general-purpose AI models in ECG analysis: A comparative study with emergency medicine specialists. *The American journal of emergency medicine* **95**, 220-226 (2025).
450. Angelaki, E., *et al.* Diagnostic performance of single-lead electrocardiograms for arterial hypertension diagnosis: a machine learning approach. *J Hum Hypertens* **39**, 58-65 (2025).
451. Díaz-Herrera, B.A., *et al.* Derivation of an artificial intelligence-based electrocardiographic model for the detection of acute coronary occlusive myocardial infarction. *Arch Cardiol Mex* **95**, 178-187 (2025).
452. Gao, Z., Yang, Y., Yang, Z., Zhang, X. & Liu, C. Electrocardiograph analysis for risk assessment of heart failure with preserved ejection fraction: A deep learning model. *ESC Heart Fail* **12**, 631-639 (2025).
453. Gupta, M.D., *et al.* Comparative evaluation of machine learning models versus TIMI score in ST-segment-elevation myocardial infarction patients. *Indian Heart J* **77**, 133-141 (2025).
454. Herman, R., *et al.* Artificial Intelligence-Powered Electrocardiogram Detecting Culprit Vessel Blood Flow Abnormality: AI-ECG TIMI Study Design and Rationale. *J Soc Cardiovasc Angiogr Interv* **4**, 102494 (2025).
455. Lee, M.S., *et al.* Artificial intelligence applied to electrocardiogram to rule out acute myocardial infarction: the ROMIAE multicentre study. *European heart journal* **46**, 1917-1929 (2025).
456. Lim, J., *et al.* Artificial intelligence-enhanced six-lead portable electrocardiogram device for detecting left ventricular systolic dysfunction: a prospective single-centre cohort study. *European heart journal. Digital health* **6**,

476-485 (2025).
457. Masumura, M., *et al.* AI-ECG for early detection of atrial fibrillation: First-year results from a stroke prevention study in Shimizu, Japan. *J Arrhythm* **41**, e70132 (2025).
458. Rudland, S.V., Shah, N.H. & Nevill, A. Community-based cardiovascular risk assessment using the Cardisio(TM) AI test: a prospective cohort study. *BJGP Open* (2025).
459. Shroyer, S., *et al.* Accuracy of cath lab activation decisions for STEMI-equivalent and mimic ECGs: Physicians vs. AI (Queen of Hearts by PMcardio). *The American journal of emergency medicine* **97**, 193-199 (2025).
460. Wang, J., Nouraie, S.M., Kelly, N.J. & Chan, S.Y. Deep learning predicts cardiac output from seismocardiographic signals in heart failure. *medRxiv* (2025).
461. Weizman, O., *et al.* Machine learning score to predict in-hospital outcomes in patients hospitalized in cardiac intensive care unit. *European heart journal. Digital health* **6**, 218-227 (2025).
462. Chow, J.S.F., *et al.* A descriptive study of the clinical impacts on COVID-19 survivors using telemonitoring (The TeleCOVID Study). *Front Med Technol* **5**, 1126258 (2023).
463. Charman, S.J., *et al.* Clinical validation of an artificial intelligence-based decision support system for diagnosis and risk stratification of heart failure (STRATIFYHF): a protocol for a prospective, multicentre longitudinal study. *BMJ open* **15**, e091793 (2025).
464. Hernandez-Hernandez, J., *et al.* Prospective study of continuous rhythm monitoring in patients with early post-infarction systolic dysfunction: clinical impact of arrhythmias detected by an implantable cardiac monitoring device with real-time transmission-the TeVeO study protocol. *BMJ open* **15**, e094764 (2025).
465. Love, C.J., *et al.* Clinical implementation of an AI-enabled ECG for hypertrophic cardiomyopathy detection. *Heart (British Cardiac Society)* (2025).
466. Yao, X., *et al.* ECG AI-Guided Screening for Low Ejection Fraction (EAGLE): Rationale and design of a pragmatic cluster randomized trial. *American heart journal* **219**, 31-36 (2020).
467. Wong, C.K., *et al.* Protocol for Home-Based Solution for Remote Atrial Fibrillation Screening to Prevent Recurrence Stroke (HUA-TUO AF Trial): a randomised controlled trial. *BMJ open* **12**, e053466 (2022).
468. Adedinsewo, D.A., *et al.* Screening for peripartum cardiomyopathies using artificial intelligence in Nigeria (SPEC-AI Nigeria): Clinical trial rationale and design. *American heart journal* **261**, 64-74 (2023).
469. Shin, T.G., Lee, Y., Kim, K., Lee, M.S. & Kwon, J.M. ROMIAE (Rule-Out Acute Myocardial Infarction Using Artificial Intelligence Electrocardiogram Analysis) trial study protocol: a prospective multicenter observational study for validation of a deep learning-based 12-lead electrocardiogram analysis model for detecting acute myocardial infarction in patients visiting the emergency department. *Clinical and experimental emergency medicine* **10**, 438-445 (2023).
470. Wijesurendra, R., *et al.* Active Monitoring for AtriaL FIbrillation (AMALFI): Rationale, protocol, and pilot for a pragmatic, randomized, controlled trial of remote screening for asymptomatic atrial fibrillation. *American heart journal* **290**, 310-324 (2025).
471. Chen, K.W., *et al.* Artificial intelligence-assisted remote detection of ST-elevation myocardial infarction using a mini-12-lead electrocardiogram device in prehospital ambulance care. *Frontiers in cardiovascular medicine* **9**, 1001982 (2022).
472. Yao, X., *et al.* Artificial intelligence-enabled electrocardiograms for identification of patients with low ejection fraction: a pragmatic, randomized clinical trial. *Nature medicine* **27**, 815-819 (2021).
473. Adedinsewo, D.A., *et al.* Artificial intelligence guided screening for cardiomyopathies in an obstetric population: a

pragmatic randomized clinical trial. *Nature medicine* **30**, 2897-2906 (2024).

474. Lin, C.*, et al.* Artificial Intelligence–Powered Rapid Identification of ST-Elevation Myocardial Infarction via Electrocardiogram (ARISE)—A Pragmatic Randomized Controlled Trial. *NEJM AI* **1**, AIoa2400190 (2024).

475. Lin, C.S.*, et al.* AI-enabled electrocardiography alert intervention and all-cause mortality: a pragmatic randomized clinical trial. *Nature medicine* **30**, 1461-1470 (2024).

476. Liu, W.T.*, et al.* Artificial Intelligence-Enabled ECGs for Atrial Fibrillation Identification and Enhanced Oral Anticoagulant Adoption: A Pragmatic Randomized Clinical Trial. *Journal of the American Heart Association* **14**, e042106 (2025).

477. Tsai, D.J.*, et al.* Artificial intelligence-assisted diagnosis and prognostication in low ejection fraction using electrocardiograms in inpatient department: a pragmatic randomized controlled trial. *BMC Med* **23**, 342 (2025).

478. Liu, W.C.*, et al.* An Artificial Intelligence-Based Alarm Strategy Facilitates Management of Acute Myocardial Infarction. *J Pers Med* **11**(2021).

479. Noseworthy, P.A.*, et al.* Artificial intelligence-guided screening for atrial fibrillation using electrocardiogram during sinus rhythm: a prospective non-randomised interventional trial. *Lancet (London, England)* **400**, 1206-1212 (2022).

480. Wang, Y.C.*, et al.* Implementation of an All-Day Artificial Intelligence-Based Triage System to Accelerate Door-to-Balloon Times. *Mayo Clinic proceedings* **97**, 2291-2303 (2022).

481. Thao, V.*, et al.* Cost-Effectiveness of Artificial Intelligence-Enabled Electrocardiograms for Early Detection of Low Ejection Fraction: A Secondary Analysis of the Electrocardiogram Artificial Intelligence-Guided Screening for Low Ejection Fraction Trial. *Mayo Clin Proc Digit Health* **2**, 620-631 (2024).

482. Hsieh, P.H.*, et al.* Economic analysis of an AI-enabled ECG alert system: impact on mortality outcomes from a pragmatic randomized trial. *NPJ Digit Med* **8**, 348 (2025).
